# Assessing Bias and Reproducibility of Viral Metagenomics Methods for the Combined Detection of Faecal RNA and DNA Viruses

**DOI:** 10.3390/v17020155

**Published:** 2025-01-23

**Authors:** Rik Haagmans, Oliver J. Charity, Dave Baker, Andrea Telatin, George M. Savva, Evelien M. Adriaenssens, Penny P. Powell, Simon R. Carding

**Affiliations:** 1 Food, Microbiome, and Health Research Programme, Quadram Institute Bioscience, Norwich Research Park, Norwich NR4 7UQ, UK; rik.haagmans@quadram.ac.uk (R.H.); oliver.charity@quadram.ac.uk (O.J.C.); evelien.adriaenssens@quadram.ac.uk (E.M.A.); 2 Norwich Medical School, University of East Anglia, Norwich NR4 7TJ, UK; 3 Core Science Resources, Quadram Institute Bioscience, Norwich NR4 7UQ, UK; david.baker@quadram.ac.uk (D.B.); andrea.telatin@quadram.ac.uk (A.T.); george.savva@quadram.ac.uk (G.M.S.); 4 Microbes and Food Science Research Programme, Quadram Institute Bioscience, Norwich Research Park, Norwich NR4 7UQ, UK

**Keywords:** viral metagenomics, mock community, eukaryotic viruses, bacteriophages

## Abstract

Whole transcriptome amplification (WTA2) and sequence-independent single primer amplification (SISPA) are two widely used methods for combined metagenomic sequencing of RNA and DNA viruses. However, information on the reproducibility and bias of these methods on diverse viruses in faecal samples is currently lacking. A mock community (MC) of diverse viruses was developed and used to spike faecal samples at different concentrations. Virus-like particles (VLPs) were extracted, nucleic acid isolated, reverse-transcribed, and PCR amplified using either WTA2 or SISPA and sequenced for metagenomic analysis. A bioinformatics pipeline measured the recovery of MC viruses in replicates of faecal samples from three human donors, analysing the consistency of viral abundance measures and taxonomy. Viruses had different recovery levels with VLP extraction introducing variability between replicates, while WTA2 and SISPA produced comparable results. In comparing WTA2- and SISPA-generated libraries, WTA2 gave more uniform coverage depth profiles and improved assembly quality and virus identification. SISPA produced more consistent abundance, with a 50% difference between replicates occurring in ~20% and ~10% of sequences for WTA2 and SISPA, respectively. In conclusion, a bioinformatics pipeline has been developed to assess the methodological variability and bias of WTA2 and SISPA, demonstrating higher sensitivity with WTA2 and higher consistency with SISPA.

## 1. Introduction

The human gastrointestinal (GI) virome consists of diverse viruses of prokaryotes (phages) and eukaryotes and plays a major role in human health and disease, with phage–host dynamics affecting the structure and function of the GI microbiome, and eukaryotic viruses directly infecting human cells [1,2,3]. Metagenomics methods enable the collective analysis of microbial genomes (i.e., including bacterial and viral genomes: the microbiome) and have been used for gastrointestinal (GI) virome analysis since the first study of the human GI virome [4]. The majority of metagenomics-based virome studies to date have focused almost exclusively on double-stranded DNA (dsDNA) bacteriophages (phages) [5]. However, recent metatranscriptomic studies [6,7] have shown that the abundance of RNA phages has been underestimated [8] and a significant portion of human viruses have RNA genomes. Therefore, comprehensive metagenomic analysis of GI RNA and DNA viruses is important for human health as it improves our understanding of the interactions between phages and bacteria, as well as the surveillance and monitoring of human GI viruses [9]. Standardised methods for which bias and reproducibility have been assessed are important, particularly for longitudinal studies, to account for any methodological variability [10,11].

Viromes derived from faecal samples are commonly used as a proxy for the GI virome. Metagenomic protocols employ nucleic acid extracted from bulk faecal homogenates or from samples enriched for virus-like particles (VLPs) to reduce the amount of non-viral nucleic acid. While VLP samples contain reduced amounts of non-viral sequences, the VLP enrichment process can affect the recovery of viruses and therefore reduce accuracy and introduce bias [5,12,13,14,15,16,17]. For example, short-term storage at 4 °C and ambient temperatures, and long-term storage at −80 °C have negligible effects on phage virome composition [17]. On the other hand, operator bias can have a significant effect on between-sample variation in virome composition [17]. As a general principle, minimal sample processing is the optimal approach to achieve optimal virus recovery. Some widely used VLP extraction and purification methods including ultracentrifugation, CsCl density gradient centrifugation, ultrafiltration, and tangential flow filtration can lead to the loss of specific viruses [12,13,14,15,16]. Once extracted, direct ligation of sequencing adapters to recovered nucleic acid without subsequent amplification produces the least bias [16,18,19] but requires quantities of input material (0.5–5 g) and/or concentration (e.g., polyethylene glycol precipitation), which is time consuming [16,17,20]. This approach is also not possible using smaller biomass faecal samples, which require amplification to achieve sufficient quantities of nucleic acid for sequencing libraries.

Popular amplification methods include multiple displacement amplification (MDA) and sequence-independent single primer amplification (SISPA), both of which can include a reverse transcription step to produce complementary DNA (cDNA) for sequencing of RNA genomes [17,21]. MDA is known to introduce amplification bias of circular genomes due to rolling circle amplification [15,22,23], and introduces other amplification biases [24,25] that cannot be overcome by pooling samples [26]. SISPA randomly amplifies DNA and RNA virus genomes using a reverse transcriptase and random primers with a 5′ universal sequence [27,28]. While SISPA produces better representation of community relative abundances, both the MDA and SISPA libraries have higher GC bias and non-uniform genome coverage compared to non-amplified libraries [15]. Whole transcriptome approaches such as Whole Transcriptome Amplification (Sigma WTA2), as implemented in the NetoVir protocol, have shown high reproducibility and good correlation of read numbers with qPCR-based virus abundance [12]. The WTA2 approach is less time consuming than SISPA, although it is costly and has been—in our experience—susceptible to supply chain issues.

A common approach to evaluating viromics pipelines is the use of specific viruses or mixtures of viruses (mock virus communities, MCs) [12,13,14,15,23,29] that are added to (spiked) faecal samples to assess the recovery and loss of viral sequences across the pipeline [13,16,17,19,30,31]. However, most of these studies exclude RNA viruses [13,16,19,23] and use different combinations of VLP extraction and amplification methods. Moreover, each study focused on distinct aspects of bias and reproducibility such as the recovery bias of MC and faecal viruses, sequencing and GC bias, over- or underrepresentation of different virus sequences, genome coverage and genome coverage depth, diversity of faecal viruses, and taxonomic variation. While WTA2 can produce a good correlation of read numbers to qPCR-based abundance of MC viruses [12], a comprehensive evaluation of other biases and reproducibility characteristics and its performance with faecal samples and its comparison with other amplification methods is lacking.

To address these issues, we have constructed a virus MC consisting of equal numbers of diverse viruses, including the dsDNA phages T5, Det7, and P22, the ssDNA phage M13, the dsDNA murid gammaherpesvirus-68 (MHV-68), dsRNA Rotavirus A (RV-A), and ssRNA viruses bovine viral diarheavirus-1 (BVDV-1). The MC was added to three faecal samples at two different concentrations, and VLPs were extracted from these spiked samples, as well as from an untreated aliquot of the sample. VLP nucleic acid was then processed using WTA2 and SISPA and sequenced. Using a custom bioinformatics pipeline, various bias and reproducibility metrics were assessed. This included recovery, sequencing depth uniformity, GC bias, and accuracy of de novo assembly-based relative abundance measurement of MC viruses. Additionally, the precision of relative abundance measurements of the faecal virome was as assessed by comparing the relative abundance between replicates and for different taxonomic clades.

## 2. Materials and Methods

### 2.1. Samples and Ethics

Three faecal samples were obtained for the study “Autoimmunity in ME/CFS” (AI-ME/CFS), registered on ClinicalTrials.gov with number NCT03254823 [32] with ethical approval obtained from the National Research Ethics Service (NRES) Committee London Hampstead (17/LO/1102, IRAS ID 218545). The samples came from healthy control individuals enrolled in the trial and were collected between March 2018 and October 2019, and were homogenised by mixing, divided into 100 mg aliquots, and stored at −80 °C as described previously [32]. Participants provided informed consent for the use of samples in subsequent research. All research was performed in accordance with the Declaration of Helsinki [33], and the International Council for Harmonisation of Technical Requirements for Registration of Pharmaceuticals for Human Use (ICH)-Good Clinical Practice (ICH-GCP) guidelines. Data were handled following the European Union General Data Protection Regulation (GDPR) and United Kingdom Data Protection Act 2018.

### 2.2. Construction of the Mock Virus Community

A virus MC was constructed using virus stocks consisting of tailed phages T5, Det7, and P22, the filamentous phage M13, and the mammalian viruses MHV-68, BVDV-1 strain Ky1203nc [34], and RV-A strain simian rotavirus SA/11 (Table 1). Virus titres were determined by epifluorescence microscopy using a protocol adapted from [35,36]. Briefly, a 13 mm Al2O3 Anodisc 0.02 μm filter membrane (Cytiva, Little Chalfont, UK, ref 6809-7003) was placed in a Swinnex filter holder (Millipore, Darmstadt, Germany, ref SX0001300) fitted onto a glass tube protruding through a rubber stopper into a Büchner flask. The flask was connected to a Millivac Maxi vacuum pump (Millipore, Darmstadt, Germany, ref SD1P014M04). After the pump was switched on, 400 μL deionised water or PBS was added to the filter holder to confirm the inlet was sealed. The stained sample was added gradually using a 1 mL micropipette. The filter was then washed with 400 μL of deionised water or PBS and any remaining liquid was aspirated for one minute. The filter was then placed sample-side-up onto Whatman filter paper (Whatman, Marlborough, MA, USA, ref 1004-055) to dry for 5 min. A 7.5 μL drop of Fluoromount G (Invitrogen, Waltham, MA, USA, ref 00-4958-02) was placed on a glass microscopy slide (VWR, Leicestershire, UK, ref 631-0117) and the filter placed on top with 7.5 μL of Fluoromount G added to the filter and covered with a glass cover slip (VWR, ref 631-0125). The slide was then left in the dark at 21 °C for at least 2 h to allow the mountant to set. The slides were imaged on an Axio Imager.M2 upright fluorescence microscope (Zeiss, Cambourne, UK). Samples were illuminated using a HAL 100 illuminator with a quartz collector 43 (Zeiss, Cambourne, UK, ref 423000-9901-000) and a 65HE Alexa 488 filter. For titre measurements, a 100X EC Plan-Neofluar oil immersion objective (Zeiss, Cambourne, UK, ref 420496-990-000) was used. Images were captured on an ICX 285 CCD monochrome camera (Sony, Surrey, UK). Between 15 and 20 images were taken of each filter at random locations.

To prepare the MC, each virus stock was diluted in PBS to obtain the same final concentration, except for MHV-68, for which the concentration was 25% of the concentration of the other viruses. A high concentration mock community (HI) was constructed such that 35 μL of the final MC contained 0.25 × 10^7^ particles of MHV-68 and 1 × 10^7^ of each of the other viruses, for a total of 6.25 × 10^7^ virus particles. A lower amount of MHV-68 was used due to limited availability of stock. As the estimated number of virus particles in human faeces is up to ~10^9^ particles/g [36], the total number of virus particles in 35 µL HI roughly corresponded to the maximum expected number of virus particles in 50 mg faeces, for a final concentration of 1.25 × 10^9^ MC particles per gram of faeces. A low concentration MC (LO) was produced by 100-fold dilution of HI in PBS, with 35 µL of LO added to a 50 mg faecal sample corresponding to 1.25 × 10^7^ MC particles per gram of faeces. MCs were stored at 4 °C.

### 2.3. Spiking of Faecal Samples

**Figure 1:**
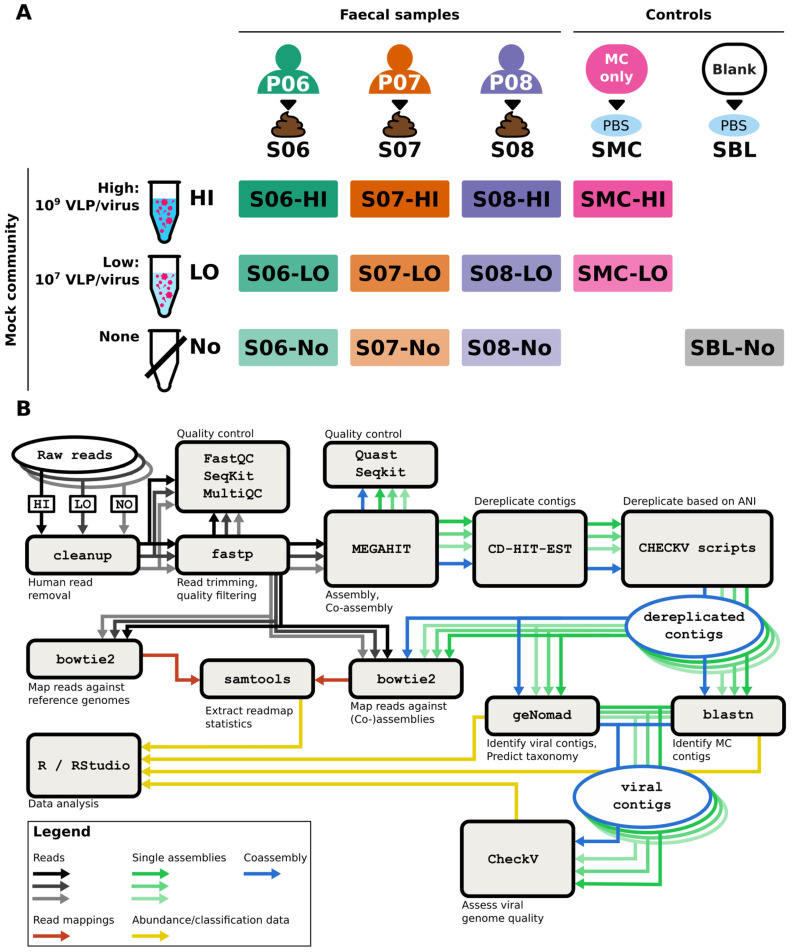
Overview of experimental design and methodology. (**A**) Stool samples (S06, S07, and S08) were obtained from three donors (P06, P07, and P08, respectively). After homogenisation, three aliquots were obtained from each stool sample. To one aliquot, a high-concentration MC (HI) was added, to the second aliquot a low-concentration MC (LO) was added, and the third aliquot was untreated (No). Additionally, an MC-only sample (SMC) of HI and LO and a blank PBS sample (SBL-No) were processed. (**B**) Bioinformatics workflow. Black and grey arrows depict flow of read data from raw reads to assembler and read mapping. Green and blue arrows indicate flow of assemblies of single samples and co-assembly of pooled samples, respectively, from the same stool sample from assembler to contig classification and read mapping. Red arrows indicate the flow of read mapping data, and yellow arrows indicate the flow of abundance and classification data to the data analysis stage.

For each faecal sample, 1.9–3.5 g was diluted to 10% (*w*/*v*) in PBS and homogenised using a Stomacher 400 Circulator Lab Blender (Seward, Worthing, UK) set to 260 RPM for 3 min. To 1 mL aliquots of S06, S07, and S08, 35 μL of HI MC was added, producing samples S06-HI, S07-HI, and S08-HI, respectively (Figure 1A). A second 1 mL aliquot of each sample was spiked with LO MC, producing samples S06-LO, S07-LO, and S08-LO, with a third control aliquot without spiking designated S06-No, S07-No, and S08-No. Additionally, 35 μL MC HI and 35 μL MC LO were each added to 1 mL PBS to produce the blank samples SMC-HI and SMC-LO, respectively, and a blank PBS sample was included, designated SBL-NO.

### 2.4. VLP Nucleic Acid Extraction

Samples were centrifuged at 16,000× *g* for 3 min at 21 °C, and the supernatant syringe filtered using a 0.45 μm syringe filter unit (Starlab, Milton Keynes, UK, ref E4780-1456) to extract VLPs. The VLP extract was stored at −80 °C for 16 h after which 700 μL was treated with a nuclease cocktail consisting of 1 μL Benzonase (Millipore, Gillingham, UK, ref E1014-5KU), 4 μL RNAse I (Thermo Fisher Scientific, Loughborough, UK, ref AM2294), 16 μL DNAse I (Thermo Scientific, Loughborough, UK, ref EN0521), 40 μL TURBO DNase (Thermo Fisher Scientific, Loughborough, UK, ref AM2238), and 125 μL of the respective 10X buffers, and incubated at 37 °C for 2 h to digest unprotected nucleic acid. The samples were then incubated at 75 °C for 1 h and 10 μL 0.5 mM EDTA (pH = 8.0) (Thermo Fisher Scientific, Loughborough, UK, ref AM9260G) was added, to deactivate nucleases. Viral nucleic acid was extracted using the QIAmp Viral RNA Mini Kit (QIAGEN, Manchester, UK, ref 52904) according to the manufacturer’s instructions, without the addition of carrier RNA. Nucleic acid extracts were stored at −20 °C.

### 2.5. Sequencing Libraries

Two approaches were tested, one using the Complete Whole Transcriptome Amplification (WTA2) kit (Sigma-Aldrich, Gillingham, UK, ref WTA2-10RXN) [12] and another using SISPA [37].

#### 2.5.1. WTA2

**Figure 2:**
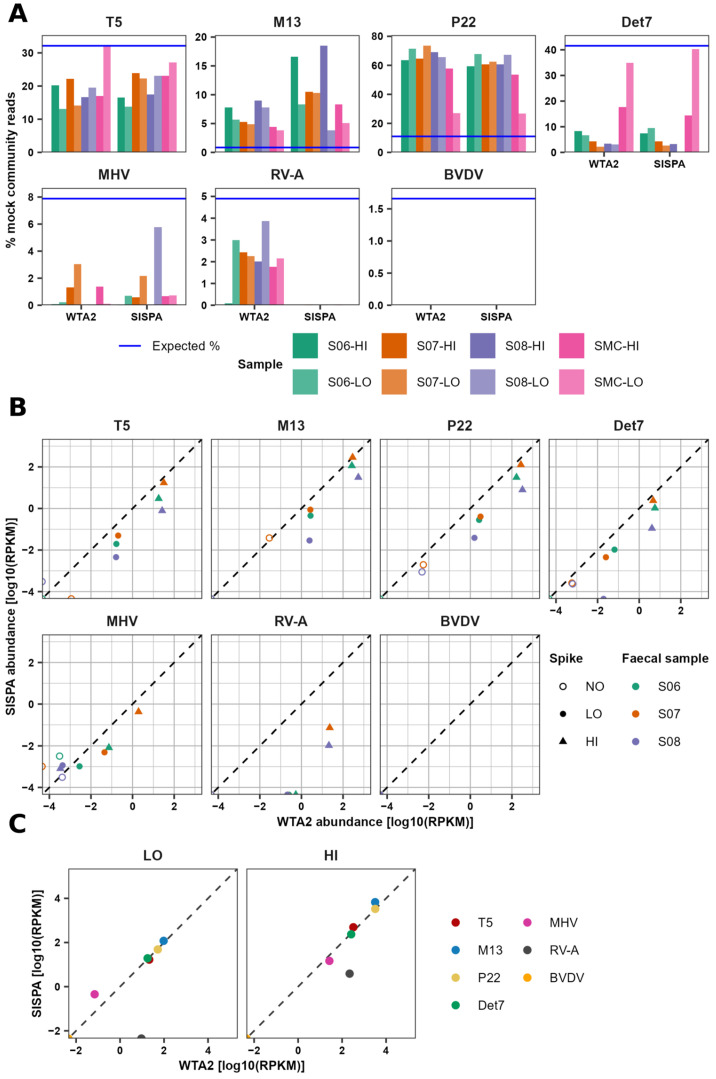
Bias and consistency of MC virus recovery in WTA2 and SISPA libraries. (**A**) Percentage of MC reads mapping to each MC virus, comparing samples processed using WTA2 to SISPA. The blue horizontal line shows the expected fraction of reads mapping to each virus based on the number of particles added, and the total genome length and number of strands. (**B**) Comparison of the abundance of each virus in the two spiked and one non-spiked aliquots of the three stool samples, generated using the WTA2 and SISPA methods. The diagonal dashed line depicts a perfect correlation. (**C**) Comparison of MC virus abundance in the MC HI control sample processed using WTA2 and SISPA. Abundance is measured in reads per kilobases of genome per million reads (RPKM).

The dsDNA pool was prepared as described previously [12]. Briefly, 2.82 μL of the sample was added to 0.5 μL of Library Synthesis Solution containing universal sequence-tagged quasi-random hexamer primers. The sample was denatured at 95 °C for 2 min and then cooled to 18 °C to prime RNA and DNA. Then, 1.68 μL library synthesis master mix was added, containing 0.5 μL Library Synthesis Buffer, 0.78 μL of RNAse-free water, and 0.4 μL Library Synthesis Enzyme and incubated in a thermocycler set to 18 °C for 10 min, 25 °C for 10 min, 37 °C for 30 min, 42 °C for 10 min, and 70 °C for 20 min, to produce a dsDNA library (Figure 2A). From each dsDNA library, 5 μL was added to 69.95 μL amplification master mix, containing 7.5 μL Amplification Mix with universal sequence primers, 60.2 μL nuclease-free water, 1.5 μL WTA2 dNTP mix, and 0.75 μL Amplification Enzyme. The sample was incubated in a thermocycler set to 94 °C for 2 min, followed by 17 cycles of 94 °C for 2 min and 70 °C for 5 min, to amplify the dsDNA fragments. DNA was then extracted using the QIAquick PCR Purification Kit (QIAGEN, Manchester, UK) according to the manufacturer’s instructions and stored at −20 °C.

#### 2.5.2. SISPA

SISPA was used to produce a dsDNA pool for sequencing from the same nucleic acid extracts processed using the WTA2 kit. Samples were processed as described previously [37]. Briefly, 4 μL of the sample was added to 9 μL master mix containing 1 μL RNasin (40 U/μL) (Promega, Chilworth Southampton, UK, ref N261A), 1 μL 10 mM dNTPs (New England Biolabs, Hitchin, UK, ref N0447S), 1 μL 20 μM primer D2_8N (5′ AAGCTAAGACGGCGGTTCGGNNNNNNNN-3′), and 6.5 μL nuclease-free water (Thermo Fisher Scientific, Loughborough, UK, ref AM9937), incubated at 65 °C and then cooled to 4 °C. Then, 6 μL master mix containing 4 μL 5X first strand buffer, 1 μL 0.1 mM dithiothreitol, and 1.0 μL SuperScript III reverse transcriptase (200 U/μL) (Thermo Fisher Scientific, Loughborough, UK, ref 18080044) was added and incubated in a thermocycler at 50 °C for 1 h for first strand synthesis. Then, 1.5 μL master mix containing 0.85 μL nuclease-free water, 0.15 μL 10X Klenow buffer, and 0.5 μL DNA polymerase I large (Klenow) fragment (5 U/μL) (New England Biolabs, Hitchin, UK, ref M0212L) was added, and the sample was incubated at 37 °C for 1 h for second strand synthesis, followed by incubation at 75 °C for 10 min to inactivate the enzyme. Free primers and nucleotides were then digested and dephosphorylated by incubation with 20 μL master mix containing 17 μL nuclease-free water, 1.0 μL 10X Shrimp Alkaline Phosphatase (SAP) buffer, 1.0 μL Exonuclease I (20 U/μL) (New England Biolabs, Hitchin, UK, ref M0293S), and SAP (1 U/μL) (New England Biolabs, Hitchin, UK, ref M0371S), respectively, at 37 °C for 1 h, followed by 15 min at 75 °C to inactivate the enzymes. The sample was then frozen at −20 °C for 16 h. The following day, the dsDNA library was generated and amplified from 8 μL of the reverse-transcribed sample, by adding it to 42 μL master mix containing 26 μL nuclease-free water, 5 μL 10X PCR buffer, 6 μL 25 mM MgCl2, 1.5 μL 10mM dNTP, 3 μL 20 μM primer D2 (5′ AAGCTAAGACGGCGGTTCGG-3′), and 0.5 μL AmpliTaq Gold (5 U/μL) (Thermo Fisher Scientific, Loughborough, UK, ref 10685095) DNA polymerase by incubation on a thermocycler. The thermocycler program consisted of: (1) denaturation for 5 min at 95 °C, (2) 5 cycles of denaturation at 95 °C for 1 min, annealing at 55 °C for 1 min, and extension at 72 °C for 1:30 min, (3) 25 cycles of denaturation at 95 °C for 30 s, annealing at 55 °C for 30 s, and extension at 72 °C for 1:30 min, adding 2 s to the extension time every cycle, and (4) final extension at 72 °C for 10 min. Samples were kept on ice between incubation steps throughout. The PCR product was loaded onto a 2% agarose (Melford, Ipswich, UK, ref 3913900099) gel in 0.5X TBE buffer (Thermo Fisher Scientific, Loughborough, UK, ref J62788.K2), and inspected for a smear between 200 and 500 bp. DNA was then extracted using the gDNA Cleanup & Concentrator-10 kit (Zymo Research, Freiburg im Breisgau, Germany, ref D4010) following manufacturer’s instructions. Purified DNA of the WTA2 and SISPA methods was quantified using a Qubit dsDNA HS Assay Kit (Thermo Fisher Scientific, Loughborough, UK, ref Q32851) on a Qubit 3.0 Fluorometer (Thermo Fisher Scientific, Loughborough, UK, ref Q33216) and samples were normalised to 5 ng/μL prior to sequencing library preparation.

### 2.6. Illumina Library Preparation and Sequencing

For preparation of the Illumina sequencing library, 0.5 µL of Tagmentation Buffer 1 was mixed with 0.5 µL Bead Linked Transposomes (Illumina, Cambridge, UK, ref 20018704) and 4 µL nuclease-free water in a master mix, and 5 μL added to a 96-well plate. A total of 2 µL of DNA normalised to 5 ng/μL was pipette-mixed with 5 µL of the Tagmentation mix and heated to 55 °C for 15 min. A PCR master mix was made using 10 µL KAPA 2G Fast Hot Start Ready Mix (Merck, Gillingham, UK, ref KK5601) and 2 µL PCR grade water per sample. Of the PCR master mix, 12 µL was added to each well to be used in a 96-well plate, and 1 µL of 10 µM primer mix containing both P7 and P5 Illumina barcodes [38] were added to each well. For the WTA2 samples, custom 9 bp dual barcodes were used, while for the SISPA samples, custom 10 bp unique dual index barcodes were used. Finally, 7 µL Tagmentation mix was added and mixed. The PCR was run at 72 °C for 3 min, 95 °C for 1 min, then 14 cycles of 95 °C for 10 s, 55 °C for 20 s, and 72 °C for 3 min. The libraries were quantified using the Promega QuantiFluor dsDNA System (Promega, Chilworth Southampton, UK, ref E2670) and measured on a GloMax Discover Microplate Reader (Promega, Chilworth Southampton, UK, ref GM3000). Libraries were pooled following quantification in equal quantities. The final pool was size selected using solid phase reversible immobilisation (SPRI) beads at 0.5X concentration, followed by SPRI beads size selection at 0.7X concentration, using Illumina DNA Prep, (M) Tagmentation sample purification beads (Illumina, Cambridge, UK, ref 20060059). The final pool was quantified on a Qubit 3.0 Fluorometer and run on a D5000 ScreenTape (Agilent, Stockport, UK, ref 5067-5579) using the Agilent TapeStation 4200 (Agilent, Stockport, UK) to calculate the final library pool molarity. The WTA2 pool was run at a final concentration of 1.8 pM on an Illumina NextSeq 500 instrument with a high-output 300-cycle flow cell (Illumina, Cambridge, UK, ref 20024908). The SISPA pool was run at a final concentration of 750 pM on an Illumina NextSeq 20000 instrument using a P3 300-cycle flow cell (Illumina, Cambridge, UK, ref 20040561). Each were run following the Illumina recommended denaturation and loading recommendations and included a 1% PhiX Control v3 spike-in (Illumina, Cambridge, UK, ref FC-110-3001).

### 2.7. Bioinformatics Analysis

An overview of the bioinformatics pipeline used is depicted in Figure 1B. The pipeline was composed in Nextflow v.24.04.3 [39]. The pipeline is available at https://github.com/RHaagmans/mc-spike (accessed 27 November 2024).

#### 2.7.1. Host Read Removal

Before running the analysis pipeline, host reads were removed using the cleanup v1.4-11-gf422ea8 Nextflow pipeline (available from https://github.com/telatin/cleanup, accessed on 17 July 2024). Briefly, host reads were removed using Kraken2 against a custom masked human reference genome [40], SARS-CoV-2 genome, and phage PhiX174 genome using Kraken2 [41] followed by adapter trimming using fastp v.0.23.4 [42,43] and a quality report generated using MultiQC [44].

#### 2.7.2. Read Quality Control

Read quality before and after quality filtering was analysed using FastQC v.0.12.1 [45]. Adapter sequences were trimmed, and reads were filtered based on quality using Fastp with the default settings and the options automatic adapter detection, base correction for pair-ended data, and cutting of the tail sequence. Fastp and FastQC outputs were gathered and visualised in MultiQC v.1.23. Duplicate reads were detected using the HTStream v1.3.3 module SuperDeduper, with default length set to 100, and the minimum read quality score set to 1. Read statistics were extracted using SeqKit v.2.8.2 [46] stats command.

#### 2.7.3. Reference-Based Relative Abundance of MC Viruses

To determine the relative abundance of MC viruses in the samples, reads were mapped against an index of mock community reference genomes (Table 2). First, reference genomes were downloaded from the National Center for Biotechnology Information (NCBI) nucleotide database using the reference genome accession numbers with NCBI Entrez Direct program efetch v.22.1, and an index was built using Bowtie 2 v.2.5.4 command bowtie2-build with default settings. Reads were then mapped to the reference index using Bowtie 2. The number of reads mapping to respective viruses was calculated using the SAMtools v.1.20 [47] idxstats command. To calculate the expected percentage of MC reads that map to each individual virus, first the number of bases each virus contributes to the sample was calculated, by multiplying the genome length by the number of added particles and the number of strands in the genome (single or double). The expected percentage of reads for each virus was calculated as a fraction of the cumulative number of bases for all viruses. MC virus abundance was calculated as the number of reads mapped to the respective genomes, per 1000 bases of genome per 1 million reads in the samples (RPKM).

#### 2.7.4. Mock Community Coverage Depth

Coverage depth and breadth of MC viruses were calculated using the SAMtools depth command with option ‘-aa’. Coverage breadth was calculated as the fraction of bases with a depth > 0. To compare sequencing depth across samples, the normalised sequencing depth was calculated as a fraction of the total number of bases sequenced for each virus in each sample.

#### 2.7.5. GC-Content of MC Reads and Genomes

The GC-content of MC reads was calculated using the SAMtools view command to extract read sequences and a custom python script to calculate the GC fraction by dividing the number of GCs in all reads mapping to a virus genome by the total number of bases mapping to the genome. Genome GC-content was calculated using the SeqKit fx2tab command.

#### 2.7.6. Assembly of Reads

High-quality reads corresponding to the same faecal sample were pooled. Pooled reads and reads from individual samples were co-assembled and assembled, respectively, using MEGAHIT v.1.2.9 [48,49] with default settings. Contigs were dereplicated in two steps. First, CD-HIT-EST v.4.8.1 [50,51] used cluster contigs based on ≥95% sequence identity, and ≥80% alignment coverage of the shorter sequence. Using BLAST v.2.15.0, a database of CD-HIT-EST was then built, and an all-versus-all alignment was performed using blastn [52]. Using the accessory scripts “anicalc.py” and “aniclust.py” from CheckV v.1.0.3 [52], contigs were again clustered, at 95% average nucleotide identity and 85% coverage of the shortest sequence to remove circularly permutated redundant sequences. Contigs were filtered from the original assembly using the list of contig IDs using the SeqKit grep command. (Co)assemblies before and after dereplication were analysed for quality control using Quast v.5.2.0 [53] and SeqKit.

#### 2.7.7. Identification and Classification of Viral and MC Sequences

For identification and taxonomic annotation of viral contigs, geNomad v.1.8.0 [54] was used with geNomad database v.1.7. Dereplicated contigs were analysed using the end-to-end pipeline in geNomad with default settings. For downstream analysis, only non-provirus contigs with a virus score > 0.8 were used. Contigs derived from MC viruses were identified using BLAST. A BLAST database was built of MC virus reference genomes and dereplicated contigs were then aligned against the MC database. Contigs that aligned with ≥98% nucleotide identity and ≥95% coverage of the contig were marked as MC sequences. Assembly genome coverage was calculated by summing the lengths of contigs matching individual viruses. Viral sequence quality was evaluated using the CheckV end-to-end pipeline with database v.1.5.

#### 2.7.8. Virus Abundance and Relative Abundance

To determine faecal viral abundances, reads were mapped against the dereplicated contigs using Bowtie 2 with default settings. Using SAMtools command idxstats, the number of reads mapping to each contig were extracted. Contig abundance was calculated on the sample and virome levels, by normalising the number of reads mapping to each contig by the contig length in kb and reads or total number of reads mapping to viral contigs, in millions (RPKM), respectively. The virome was defined as those contigs identified as viral by geNomad, excluding mock community sequences to maintain consistency between replicates with different spike-in treatments. Relative abundance was then calculated as a fraction of the cumulative total and virome abundance, respectively.

#### 2.7.9. Variation in Relative Abundance and Rank

To calculate the variation in abundance, relative abundance, and abundance ranking, reads from each of the individual replicates were mapped to the respective co-assemblies. Abundance and relative abundance were calculated on the sample and virome levels as described above. Contigs with at least one read in each of the replicates were included in the analysis. The range in abundance was calculated as the log2-transformed ratio of the highest and lowest abundance of each contig in three replicates. Abundance rank was calculated by sorting contigs by their abundance from highest to lowest in each replicate, and the range was determined as the difference between the highest and lowest ranking of a contig among the three replicates. The coefficient of variation in relative abundance was calculated as the coefficient of variation of the relative abundance of the contig in the three replicates.

#### 2.7.10. Virome Taxonomic and Diversity Analysis

Alpha diversity based on assemblies and co-assemblies was calculated using the R package phyloseq v.1.48 [55]. Beta diversity was calculated using the R package vegan v.2.6-6.1, by calculating the Bray–Curtis dissimilarity between pairs of the HI, LO, and NO replicates of each faecal sample based on the co-assembly data.

#### 2.7.11. Data Analysis and Visualization

Data were analysed in R v.4.4.1 with RStudio v.2023.06.1. Data were handled using tidyverse v.2.0.0 [56] packages, including dplyr v.1.1.4, readr v.2.1.5, forcats v.1.0.0, stringr v.1.5.1, tibble v.3.2.1, tidyr v.1.3.1, and purr v.1.0.2. Data were visualised using ggplot2 v.3.4.4 [57], ggExtra v.0.10.1 [58], ggpubr v.0.6.0 [59], ggsci v.3.0.0 [60], ggh4x v0.2.6 [61], and gt v.0.11.0 [62].

## 3. Results

To assess differences in recovery efficiency between viruses, compare recovery of individual viruses between samples, determine an appropriate MC spike-in concentration, and assess sequence bias and faecal virome variation, three faecal samples were split into three aliquots each. One aliquot of each sample was spiked with HI, another was spiked with LO, and the last aliquot was not spiked. Viral nucleic acid extracts of each sample were processed using the WTA2 and SISPA methods for RT and PCR amplification of nucleic acids and sequenced. Sequencing of WTA2 and SISPA samples returned an average of 12 million reads per sample (Table S1). A custom bioinformatics pipeline was employed to analyse the sequencing data, including removal of host sequences, quality filtering of reads, assembly of reads into contigs, and classification of contigs (Figure 1B). After quality control, an average 3.6% of reads were removed (Figure S1). WTA2 and SISPA returned equivalent numbers of reads for most samples. However, mapping high-quality reads to MC reference genomes (Table 2) in spiked faecal samples showed a 2- to 40-fold higher fraction of MC virus reads in WTA2 samples compared to SISPA (Table 3). In faecal samples spiked with HI, an average of 3.1% of reads in the WTA2 samples and an average of 0.72% reads in the SISPA samples mapped to MC viruses. In the HI- and LO-only samples, the percentage of MC reads was similar between WTA2 and SISPA libraries. In the blank sample (SBL), ~0.5% of total reads mapped to MC reference sequences, with >98% of those reads mapping to the four phages in WTA2 and SISPA libraries. Mapping of reads of the blank samples to the 2024-09-04 PlusPFP database (https://benlangmead.github.io/aws-indexes/k2 (accessed on 3 November 2024)) resulted in 17.8% and 23.8% of reads classified, with 83.8% and 87.1% of those reads mapping to bacteria in the WTA2 and SISPA library, respectively.

### 3.1. Recovery Bias and Consistency of Mock Virus Community

To assess the recovery bias of individual MC viruses, the percentage of mapped MC reads that map to each of the individual viruses was calculated and compared to the expected percentage of reads based on the number of particles of each virus added, its total genome length, and number of strands. Phage levels were closest to the expected level in the SMC (MC with PBS) samples, particularly in the SMC-LO sample (Figure 2A). The smallest fold difference between the expected and mapped fraction of reads in WTA2 and SISPA SMC samples was 0.00, 3.52, 1.45, and 0.03, whereas the smallest fold difference in spiked samples was 0.25, 3.55, 4.39, and 0.77 for phages T5, M13, P22, and Det7, respectively. Nonetheless, there were large differences between the observed and expected levels of all viruses in all samples. Phages M13 and P22 were detected up to 22 and 7 times higher, relative to the rest of the community, than expected, while all other viruses were estimated to have a relative abundance lower than expected. Levels of the enveloped eukaryotic viruses MHV-68 and BVDV-1 were lowest, particularly BVDV-1, for which no reads were detected. The lower titre of MHV-68 in the MC may have contributed to the variation observed between samples. Although MC virus levels relative to the total MC abundance were generally the same between samples and when comparing WTA2 and SISPA, there was no systematic effect visible of the faecal samples and variation is likely due to sampling and measurement error. Exceptions were M13, which was increased, and RV-A, which was virtually absent, in SISPA libraries.

To investigate the contribution of library preparation methods to the observed variation, the abundance of MC viruses in WTA2 and SISPA libraries was compared. Overall, abundance relative to the total number of reads was higher in WTA2 libraries than in SISPA libraries, and variation between samples was lower (Figure 2B). Comparing the abundance of the viruses in replicates spiked with HI and LO indicated a linear relationship and around a 100-fold difference between the two MCs. Some virus reads were also detected in the non-spiked aliquots, possibly indicating erroneous mapping, or mapping of reads from conserved regions of related faecal viruses. Comparing the abundances of the MC viruses in the HI-only sample WTA2 and SISPA libraries showed that abundances of all viruses, except RV-A and BVDV-1, were in close agreement (Figure 2C). In the SISPA library, RV-A abundance was lower, and BVDV-1 was not detected. Discrepancies between stock virus genome sequences and the reference sequences could have contributed to differences between expected and observed virus levels. However, substitution of reference sequences for metagenome assembled sequences did not have any meaningful effect.

### 3.2. Sequencing Bias in WTA2 and SISPA Libraries

**Figure 3:**
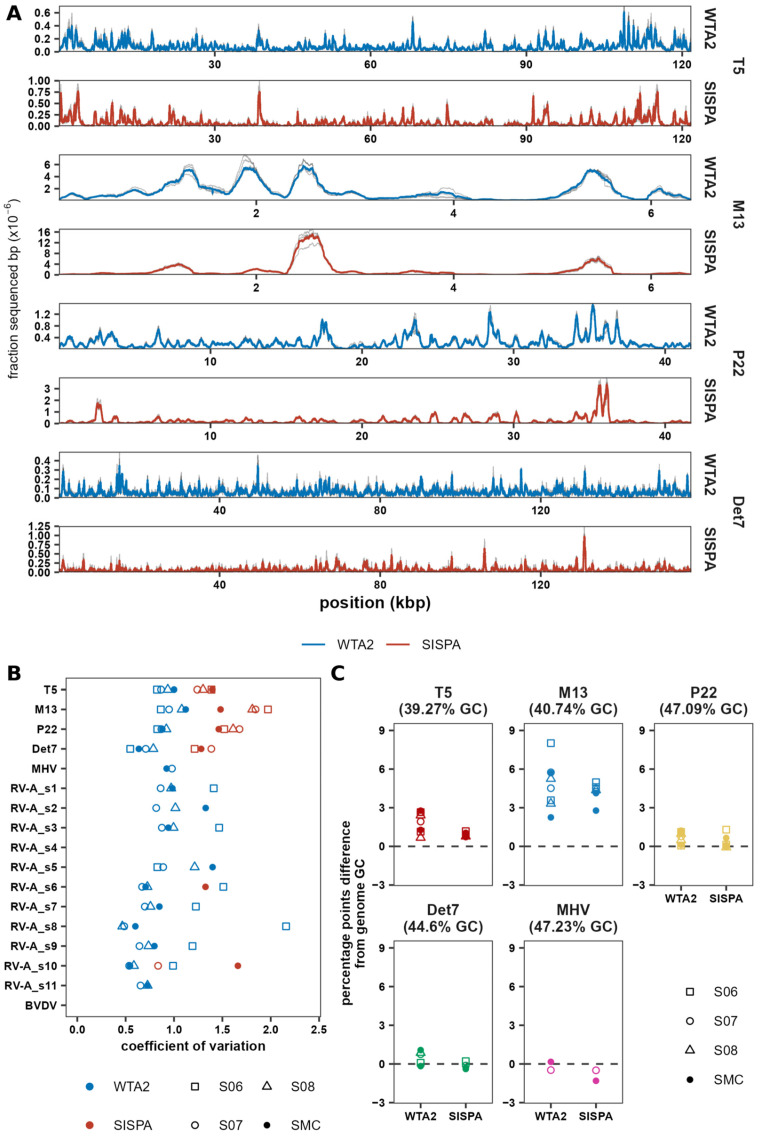
Sequencing depth uniformity and GC bias differ between WTA2 and SISPA. (**A**) Comparison of the normalised sequencing depth of T5, M13, P22, and Det7 in WTA2 and SISPA libraries. The grey lines show the normalised depth of samples in which at least 80% coverage was achieved. Thick blue and red lines depict the means of those samples. (**B**) Coefficient of variation of sequencing depth in SMC-HI and samples spiked with MC HI. (**C**) Difference in the average GC-content of reads mapping to reference genomes of viruses with a non-segmented genome, compared to the actual GC-content of the virus genome, for samples with >20% genome coverage.

Library preparation methods differ in the uniformity of genome coverage, and higher coverage uniformity reduces the sequencing depth required to recover a full genome. Differences in uniformity may therefore affect detection of viruses, particularly when depending on de novo assembly. Thus, the coverage uniformity of MC virus genomes was investigated in samples for which at least 80% of the genome was covered. For T5, P22, Det7, and M13, greater than 80% coverage was obtained in the WTA2 and SISPA libraries of at least two samples. Additionally, greater than 80% coverage was obtained for MHV-68 and segment 1 of RV-A in the WTA2 libraries for two samples. In both the WTA2 (Figure S3) and SISPA (Figure S4) libraries, the coverage depth profile, although highly non-uniform, was consistent between replicates. Coverage depth profiles of WTA2 and SISPA showed some similarity in the high-depth regions, but higher peaks were observed in the SISPA libraries. (Figure 3A). This was reflected in the coefficient of variation (CV) of the coverage depth of MC virus genomes in the WTA2 and SISPA libraries, with a mean CV for Det7, P22, T5, and M13 in all samples of 87% in the WTA2 libraries and 160% in the SISPA libraries (Figure 3B). The CV was higher for WTA2 than SISPA only for RV-A segments 6 and 10 in sample S06.

As various genomic features including GC-content can affect sequencing efficiency, the average GC-content of virus reads was compared to the genomic GC-content in samples with >20% genome coverage, as above that threshold the read GC-content did not depend on coverage (Figure S5). For DNA viruses T5 and M13 with a genomic GC-content of <41%, the mean read GC-content was higher than the virus genome GC-content (Figure 3C), while for phages P22 and Det7, the GC-content was close to the actual genomic GC-content, and for MHV-68, with the highest genomic GC-content (47%), the read GC-content was below the genomic GC-content. For all DNA viruses, the read GC-content was more consistent between replicates for the SISPA libraries than the WTA2 libraries. For the dsRNA virus RV-A, the genomic GC-content of genome segments varied from 28.9% to 40.2%. In the case of the WTA2 libraries, for the segments with a GC-content <36%, the read GC-content in most replicates was equal to or higher than the genomic GC-content (Figure S6). On the other hand, for the segments with a GC-content >36%, the read GC-content was equal to or lower than the genomic GC-content in most samples. In the case of the SISPA libraries, only two segments were sequenced to sufficient coverage and no consistent pattern could be determined.

### 3.3. Assembly Quality of WTA2 and SISPA Libraries

**Figure 4:**
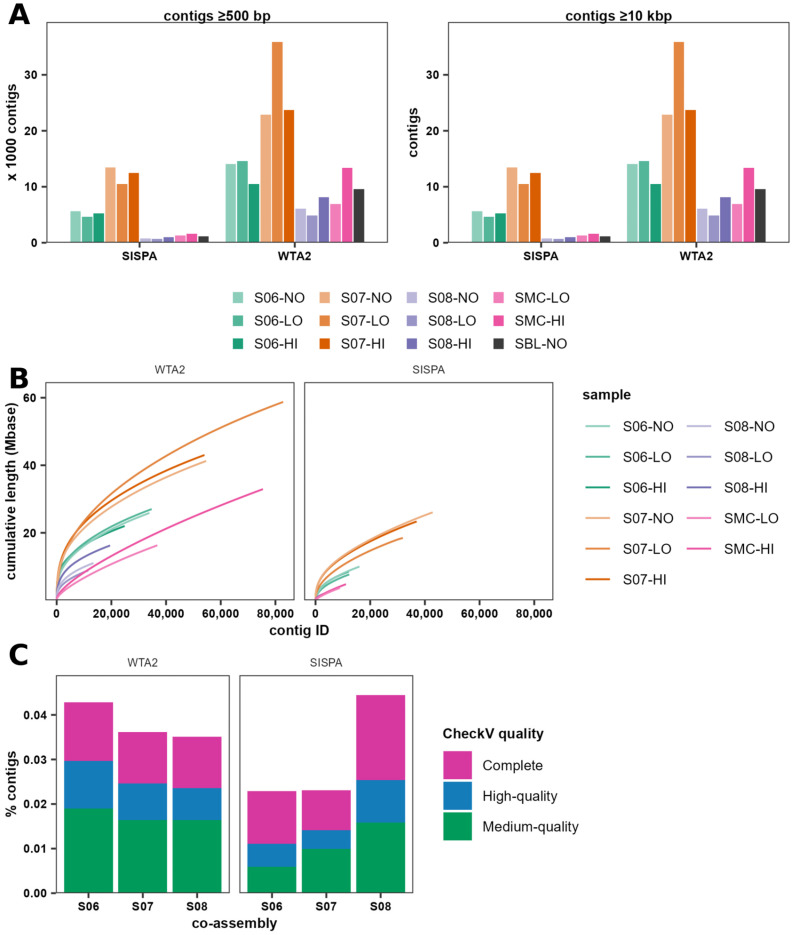
WTA2 samples yield more and larger contigs than SISPA. (**A**) Total number of contigs and number of contigs ≥10 kbp in single assemblies of each of the samples after removing redundant sequences. (**B**) Cumulative assembly length of single assemblies. (**C**) Percentage of contigs in WTA2 and SISPA co-assemblies with representing complete, high-quality, and medium-quality genomes according to CheckV.

To determine differences in assembly quality between WTA2 and SISPA, reads from individual libraries were assembled. Assembly of WTA2 libraries consistently yielded more contigs overall and more contigs >10 kb (Figure 4A). The number of contigs in the assemblies of the spiked sample SISPA libraries was between 22% and 81% lower than the WTA2 library co-assemblies. The N50 was higher in every case for WTA2 than for SISPA library assemblies, indicating a higher overall quality of assemblies (Table S2). Additionally, the largest contig in samples S06-HI and S08-HI were 125 kb and 100 kb in the WTA2 co-assemblies and 77 kb and 33 kb in the SISPA assemblies, respectively. Cumulatively, WTA2 assemblies contained 8.8–58.7 Mbases, while SISPA assemblies contained 1.4–26.0 Mbases and the cumulative length of SISPA assemblies was 37–88% lower than WTA2 libraries (Figure 4B). The average length of the 250 longest contigs in the WTA2 assemblies was between 1.9 and 4.9 times longer than SISPA assemblies, suggesting increased fragmentation and reduced diversity in the SISPA library assemblies. On the other hand, the mean coverage depth of contigs in the SISPA assemblies was between 1.6 and 18.0 times higher than WTA2 library assemblies and the overall GC-content of assemblies was more consistent in SISPA replicate libraries of the same faecal sample than in WTA2 libraries (Table S2).

Next, geNomad [54] was used to identify viral sequences, while MC sequences were identified by aligning sequences to MC reference genomes. In assemblies and co-assemblies of both WTA2 and SISPA libraries, a large fraction of contigs could not be identified as viral, indicating the presence of non-viral nucleic acid and potentially false negatives. Classification of reads mapping to these contigs using Kraken2 with the 2024-09-04 PlusPFP database determined an enrichment of bacterial reads, suggesting a bacterial origin for these contigs, with the fraction of bacterial reads increased in SISPA libraries compared to WTA2 libraries for each of the samples (Figure S8). Across faecal samples and replicates, and ignoring mock community contigs, 3.4% and 3.5% of contigs were classified as viral in WTA2 and SISPA assemblies, respectively. The relative abundance of the virus sequences was calculated by mapping reads of individual samples to the contigs of the respective assemblies. Faecal viruses contributed on average 33.1% and 6.3% of the relative abundance in the WTA2 and SISPA libraries, respectively. While SISPA assemblies had a higher proportion of MC virus contigs, the cumulative length of MC contigs was lower, indicating increased fragmentation of MC virus genomes. The collective abundance of MC contigs relative to all viral contigs was similar for both WTA2 and SISPA in samples spiked with HI, of between 0.7% and 4.6%. On the other hand, the relative abundance of contigs not identified as viral was higher in the SISPA (93%) than the WTA2 (83%) assemblies. Finally, while WTA2 (co-) assemblies had a higher fraction of viral contigs, a higher fraction of co-assembly contigs were of medium quality or higher as well for samples S06 and S07 (Figure 4C). Although a higher fraction of viral sequences in the SISPA co-assembly of sample S08 were complete genomes, the total number of complete genomes was lower in the SISPA assembly (*n* = 6) than the WTA2 assembly (*n* = 21). Together, these data suggest increased fragmentation of SISPA assemblies leading to reduced identification of virus sequences.

### 3.4. Accuracy of Assembly-Based Virus Abundance

**Figure 5:**
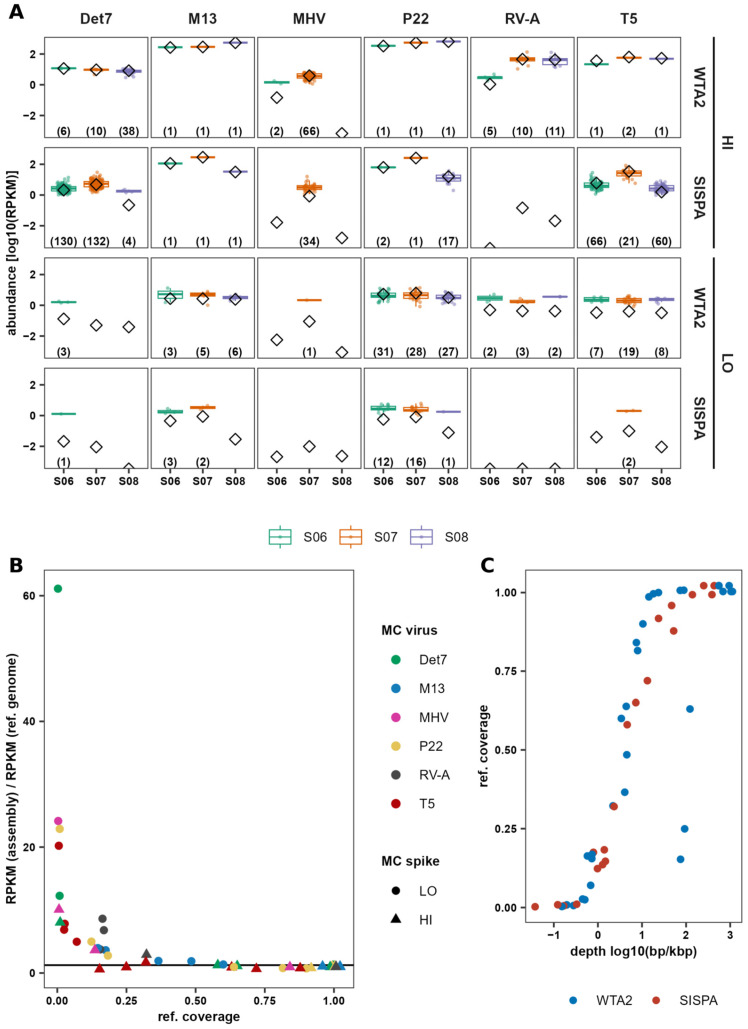
Assembly-based abundance is overestimating actual abundance when contig coverage of the genome is low. (**A**) The boxplots show the abundance of MC virus contigs in the assembly calculated as RPKM, which is compared to the calculated virus abundance based on reads mapping to the reference genomes in RPKM (diamonds). Numbers along the *x*-axis represent the number of contigs in the assemblies. (**B**) Fold difference between the weighted average abundance of MC virus contigs [RPKM (assembly)] and the MC virus abundance based on read mapping to the reference genome [RPKM (ref. genome)], compared to the total genome coverage of MC virus contigs (ref. coverage). The horizontal line shows a ratio of 1.25. (**C**) Percentage of the reference genome covered by genome fragments in the assemblies (ref. coverage) as a function of the sequencing depth. The depth is calculated from the number of sequenced bases per thousand base pairs of genomes.

To investigate the accuracy of relative abundance estimates based on de novo assembly, the relative abundance of MC contigs was compared to the MC virus relative abundances calculated using the reference genome sequence. In the assemblies of WTA2 libraries, samples spiked with HI yielded between 1 and 38 MC contigs for phages Det7, P22, and M13 (Figure 5A), all collectively covering >98% of the reference genome, while phage T5 contigs covered 15%, 63%, and 25% of the genome in samples S06, S07, and S08, respectively. For phages T5, Det7, and P22, the SISPA libraries yielded more contigs, while genome coverage of contigs was lower for T5 and Det7. Coverage for Det7 varied between 1.0% and 65%, indicating reduced fragmentation in the WTA2 library assemblies.

In the samples spiked with LO, assemblies contained more contigs for each virus, together covering a lower portion of the genome, showing higher fragmentation of the genome in these assemblies. Additionally, several samples did not yield any contigs for Det7, M13, MHV-68, RV-A, and T5 in the WTA2 and SISPA libraries. This suggests reliable assembly-based detection of viruses using this method requires >10^5^ particles in 50 mg of a faecal sample. For both the WTA2 and SISPA libraries, contigs of LO sample assemblies collectively covered lower portions of the reference genomes than those of HI sample assemblies.

Among genome fragments matching the same genome, there were considerable differences in relative abundance. Across libraries, there was up to a 24-fold difference between the fragment with the highest and lowest abundance. Calculating the virus abundance using collections of genome fragments (RPKM_assembly_) only produced estimates close to the reference genome-based abundance (RPKM_ref_) if the fragments collectively covered a sufficient portion of the reference genome (Figure 5B). When contigs collectively covered ≥80% of the reference genome, the RPKM_assembly_ was close to the RPKM_ref_ with on average 6% higher estimated abundance compared to RPKM_ref_. However, abundance was considerably overestimated when contigs covered a smaller portion of the reference genome (Figure 5B). At a maximum overestimation of 100%, 50%, and 10%, contigs collectively covered at least 32%, 58%, and 63%, respectively, of the genome.

As the sequencing depth of the virus genomes was not uniform (Figure 3A), some areas of the genome have a higher probability of producing reads and thus assembling into contigs. Indeed, WTA2 libraries have a more uniform sequencing depth and have a slightly higher coverage for the same number of sequenced bases than SISPA libraries (Figure 5C). Thus, for a given sequencing depth, a more uniform depth profile will lead to increased coverage of the genome, whereas for a more heterogeneous depth profile, reads will accumulate on a shorter area of the genome (Figure S7), which inflates the estimated abundance. This is also supported by the greater number of duplicate reads in SISPA libraries compared to WTA2, with at most 14% of reads duplicated in WTA2 libraries, against 29% in SISPA (Figure S9).

### 3.5. Consistency of Assembly-Based Faecal Virus Abundance

**Figure 6:**
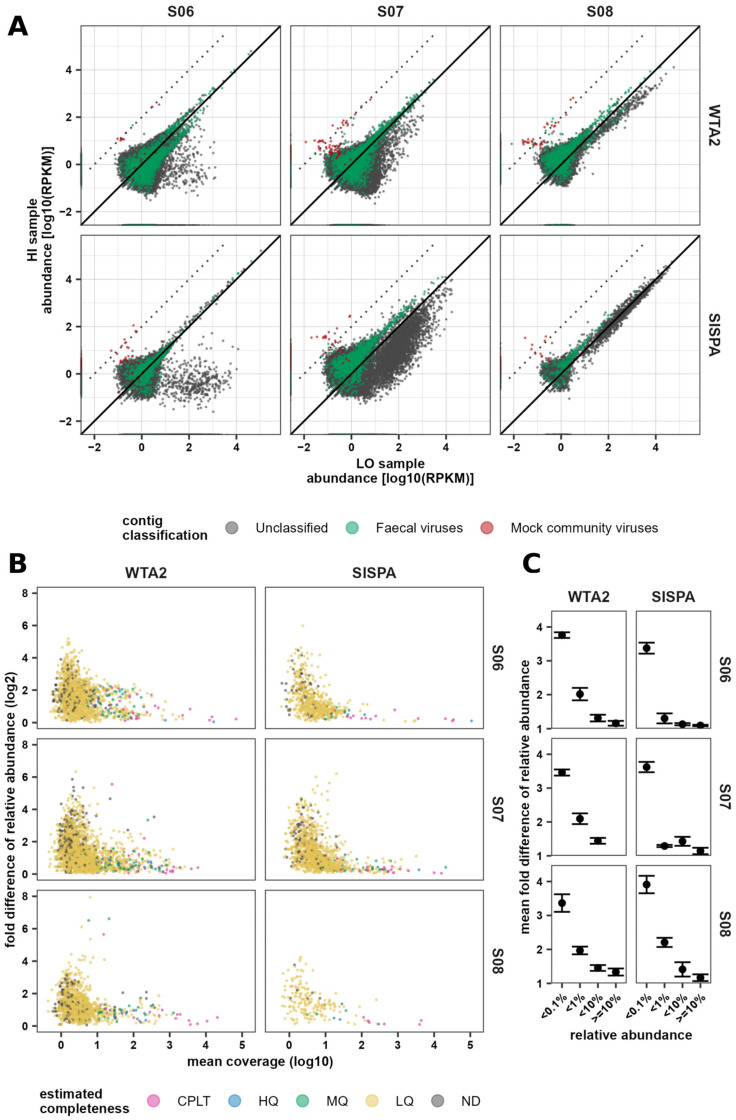
Concordance in virus abundance estimates between replicates within faecal samples is highest for high abundance contigs and medium- to high-quality genomes. (**A**) Comparison of normalised read counts (RPKM) of stool sample spikes with MC HI and LO. The black solid line depicts perfect agreement. The black dotted line indicates a 100-fold increase in the HI sample over the LO sample. (**B**) Log2 fold difference between the highest and lowest relative abundance of viral contigs across the three replicates, compared to mean coverage depth. (**C**) Variation in relative abundance measured as the mean fold difference between the highest and lowest relative abundance of contigs binned by a mean relative abundance <0.1%, between ≥0.1% and <1% (<1%), between ≥1% and <10% (<10%), and ≥10% (≥10%).

We next sought to investigate the consistency of the recovery of faecal viruses. For this, co-assemblies were generated of the three aliquots (spiked with HI, LO, and untreated) of each of the faecal samples. For this analysis, these aliquots were regarded as technical replicates as the spiking treatment was assumed to not affect the faecal virome. The abundance of faecal virus contigs in each of the replicates was determined by mapping the reads of the individual replicates to the respective co-assemblies. Comparing the abundance of viral contigs between HI and LO replicates, variation in estimated abundance decreased with increasing abundance (Figure 6A), as would be expected. In samples S06 and S07, a large group of unclassified contigs were present at 10- to 100-fold higher concentrations in the LO replicate. This difference was particularly pronounced in the SISPA libraries of sample S07. MC virus relative abundance in the HI replicate was around 100-fold higher than in the LO replicate, consistent with the difference in concentration between the MC spike-ins. To analyse the variation in abundance between replicates, only viral contigs, but not MC contigs, were subsequently used.

To analyse the variation in estimated abundance for each contig between samples, the abundance of contigs that were present in all three replicates was compared between the HI, LO, and No replicates and the fold difference between the highest and lowest abundance was calculated. While longer contigs tended to have higher coverage depth, the variation in abundance was limited mostly by coverage depth, not contig length (Figure S9). Nearly all contigs with at least a 4-fold difference between the highest and lowest abundance had a mean coverage depth of <10X. For contigs with ≥10X coverage, the median ratio between the highest and lowest abundance was 1.7 and 1.4 for the WTA2 and SISPA co-assemblies, compared to 2.5 and 2.4 for contigs with <10X coverage, respectively. Contigs with a mean coverage depth <10X will be referred to as low coverage depth (LCD), while contigs with a mean coverage depth ≥10X will be referred to as high coverage depth (HCD).

Analysing the fold difference in relative abundance between the highest and lowest abundance of contigs between replicates showed that 83% and 74% of contigs were LCD contigs in WTA2 and SISPA co-assemblies, respectively. Comparing the fold difference of LCD and HCD contigs showed a median of 2.5 for LCD contigs in WTA2 and SISPA samples, compared to 1.7 and 1.5 HCD contigs of WTA2 and SISPA samples, respectively (Figure 6B). Thus, while WTA2 co-assemblies produced more HCD contigs than SISPA co-assemblies, HCD contigs on average had higher variation in WTA2 library co-assemblies. Ranking contigs based on abundance and comparing the ranking between replicates showed a similar pattern, with the difference between highest and lowest ranking greatly increased for LCD contigs (Figure S10).

While variation was largest for LCD contigs, they collectively only contributed between 0.37% and 2.4% of faecal viral reads across all libraries, corresponding to between 1.00% and 10.5% of relative abundance. Across replicates, LCD contigs represented 3.1%, 9.6%, and 5.4% viral relative abundance in WTA2 libraries and 1.2%, 5.1%, and 5.2% in SISPA libraries for samples S06, S07, and S08, respectively. Additionally, all contigs with a relative abundance ≥0.1% were HCD contigs. Correspondingly, the range in abundance-based ranking was at most four places in both the WTA2 and SISPA libraries for contigs with an average relative abundance ≥1%, except WTA2 sample S07. Additionally, there was a median 1.5- and 1.3-fold difference between the highest and lowest relative abundance in the WTA2 and SISPA libraries, respectively, for contigs with ≥1% average relative abundance (Figure 6C). This means that for the higher abundance contigs, the abundance ranking of contigs with a relative abundance ≥1% is consistent across replicates. Nonetheless, there was up to a 50% difference between the highest and lowest relative abundance, from only three replicates, indicating considerable uncertainty in quantitative abundance estimates even in the best case. Most contigs that qualified as complete, high quality, or medium quality were HCD contigs, with 99%, 94%, and 81% contigs, respectively, having HCD. In SISPA co-assemblies, a higher fraction of medium-quality contigs were HCD contigs (88%) than in WTA2 co-assemblies (79%). Nonetheless, even among contigs of medium quality and above, the fold difference of some contigs was high, particularly in the WTA2 co-assemblies. Only 50%, 42%, and 37% of complete, high-quality, and medium-quality contigs, respectively, had at most a 1.5-fold difference in WTA2 samples, versus 92%, 93%, and 59% of those contigs, respectively, in SISPA samples. Taking all HCD contigs, two randomly selected relative abundances from the three replicates were at most 50% apart only 73.0% and 83.4% of the time in the WTA2 and SISPA libraries, respectively (Table S3), and measurements were at most 10% apart for only 15.7% and 31.1% of the time in the WTA2 and SISPA libraries, respectively. This suggests that if a difference of more than 50% abundance is measured between two samples, this could be due to sampling and measurement error alone around 30% and 20% of the time, respectively, for WTA2 and SISPA. Furthermore, the observed difference in abundance was 140% for WTA2 and 110% for SISPA in fewer than 5% of measurement pairs, suggesting that differences greater than this can be more reliably attributed to genuine differences between samples instead of measurement error.

### 3.6. Taxonomic Analysis and Sample Diversity

**Figure 7:**
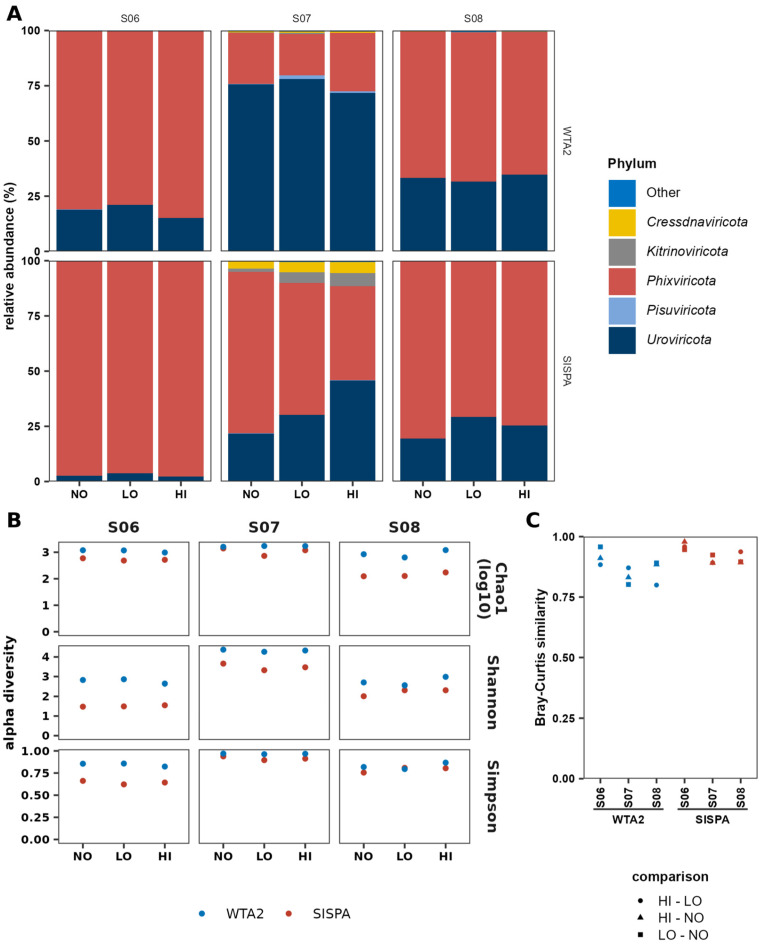
Difference in taxonomy and diversity between WTA2 and SISPA. (**A**) The five phyla with the highest relative abundance in any of the samples are shown. (**B**) Sample alpha diversity, as calculated by the Chao1 index for species richness and the Simpson and Shannon indices for species evenness. (**C**) Beta diversity, calculated through the Bray–Curtis similarity index of the replicates of each stool sample.

Lastly, the taxonomic content of the individual sample assemblies (Figure 7A) of the WTA2 and SISPA libraries was determined using the taxonomic classifications generated by geNomad, excluding MC sequences. Virtually all contigs that were identified as viral could be classified at the class level, although the classification rate at lower clades was considerably lower, with on average only 10.7% and 10.1% of viral sequences classified at the order and family levels, respectively. Overall, the phyla of *Phixviricota*, *Uroviricota*, *Kitrinoviricota*, *Cressdnaviricota*, and *Pisuviricota* were the five most abundant among all samples in both libraries. The phylum *Phixviricota* contains phages with small protein capsids and small circular ssDNA genomes, while *Uroviricota* are tailed phages with large dsDNA genomes. While phages of the phylum *Phixviricota* were the most abundant viruses in samples S06 and S08 for both WTA2 and SISPA libraries, abundance of *Phixviricota* was greater in the SISPA libraries than WTA2 libraries. For sample S07, the difference between the libraries was much greater, with a mean relative abundance of 22.7% and 58.4% in the WTA2 and SISPA libraries, respectively (Table S4). Members of the phyla *Pisuviricota* (ssRNA(+) genomes), *Cressdnaviricota* (circular ssDNA genomes), and *Kitrinoviricota* (ssRNA) infect eukaryotic cells. In sample S07, *Kitrinoviricota* and *Cressdnaviricota* were more abundant in the SISPA library than the WTA2 library. *Kitrinoviricota* and *Cressdnaviricota* had 4.1% and 4.3% relative abundances, respectively, in the SISPA library, while the WTA2 libraries of sample S07 contained 0.28% and 0.55% of these phyla, respectively. For *Cressdnaviricota*, the pattern was reversed in samples S06 and S08, with 0.067% and 0.16% relative abundances in the WTA2 library, and 0.014% and 0.028% in the SISPA libraries, respectively. Such large differences were not found for *Kitrinoviricota* in the other two faecal samples. Lastly, the phylum *Pisuviricota* had a consistently higher relative abundance in the WTA2 libraries than the SISPA libraries of all three faecal samples.

The ratio between the highest and lowest relative abundance was lowest for the exclusively prokaryotic virus phyla, with 1.05–1.42 and 1.02–1.71 for *Phixviricota* and 1.09–1.40 and 1.51–2.10 for *Uroviricota* in the WTA2 and SISPA libraries, respectively. For the largely eukaryotic virus phyla *Pisuviricota*, *Cressdnaviricota*, and *Kitrinoviricota*, the ratio was higher, and the ratio could not always be determined due to their absence in at least one of the replicates. The relative abundance of these phyla was <0.1% in most samples, and the variation was between 0.0027 and 4.3 percentage points. Thus, the relative abundance of the prokaryotic phyla *Phixviricota* and *Uroviricota*, which typically represent the bulk of the virome, are robust, compared to *Pisuviricota*, *Cressdnaviricota*, and *Kitrinoviricota*, which is likely due to the low relative abundance of these taxa in the samples.

To determine differences in virome richness between WTA2 and SISPA, and variation between replicates, the Chao1 index was used and showed a greater richness in the WTA2 libraries than the SISPA libraries (Figure 7B). The mean CV of species richness in the WTA2 and SISPA libraries was 16% and 20%, respectively. Alpha diversity was measured by the Shannon and Simpson indices with WTA2 libraries having greater evenness than the SISPA libraries. Using the co-assemblies of each the WTA2 and SISPA libraries of the base faecal samples, the Bray–Curtis similarity index was calculated between each of the replicates to determine the variation in overall sample composition between replicates of each faecal sample (Figure 7C). For all three faecal samples, similarity was higher in the SISPA libraries than in the WTA2 libraries, while the samples with the highest and lowest similarity were the same in the WTA2 and SISPA co-assemblies. Interestingly, the level of similarity corresponded to the percentage of LCD contigs in each co-assembly.

## 4. Discussion

We have assessed the bias and reproducibility of virus metagenomes obtained using two widely used methods of transcriptome amplification, WTA2 and SISPA, using an MC of diverse viruses to spike human faecal samples. In comparing the recovery of individual viruses, genome coverage depth profiles, contigs and assemblies, coverage depth and relative abundance of viral sequences, and taxonomy across replicates, WTA2 provides higher sensitivity, at the cost of higher variability, whereas the SISPA method, by comparison, is less sensitive and accurate but is more consistent. While WTA2 is a proprietary kit, SISPA uses commonly used reagents and is thus less costly and less sensitive to supply chain issues with reagents being substituted more easily.

### 4.1. Recovery of Individual Spiked Viruses from a Mock Community

The level of recovery of individual MC viruses varied between samples, which is larger for Det7 and MHV-68 than for T5 and P22. Overall, patterns were comparable between the SISPA and WTA2 libraries. Differential recovery across faecal samples of MC viruses could be due to differences in the faecal sample composition. For example, some phage capsids contain Ig-like domains that help attach to the intestinal mucus [63], including phages T5 [64] and P22 [65]. Differences in, for example, mucus content, could produce different interactions between viruses and faecal material that affect downstream recovery VLPs. Moreover, inherent differences in individual faecal samples may affect aggregation or adherence to plasticware and filter membranes, which is suggested when comparing the percentage of Det7 and P22 reads of the MC-only samples to the spiked samples (Figure 1A), with the levels in the MC-only samples being much closer to the expected value. Additionally, differences between replicates may be exaggerated in Figure 1A, as variation in the percentage of reads mapping to one virus will automatically affect the percentage of reads mapping to all others. Moreover, for the samples spiked with LO, around 100-fold fewer reads mapped to MC viruses and were more likely to be affected by noise.

In the WTA2 libraries of spiked samples, the difference between MC phage relative abundances in samples spiked with HI and LO reflected the difference in concentration of those viruses in HI and LO, in line with previous results [12]. However, the number of reads corresponding to eukaryotic viruses in the WTA2 libraries and all viruses in LO-spiked sample SISPA libraries was low and relative abundances did not reflect the difference between HI and LO in these cases. This is likely a result of increased noise associated with low read counts.

Individual viruses also differ in their recovery efficiency, with the abundance of M13 and P22 far exceeding expected levels. Phage concentrations were determined using epifluorescence microscopy, as in a previous study by Kleiner et al. [13]. They also found an increased recovery of P22 [13], suggesting a higher nucleic acid extraction efficiency of this phage. Differences in nucleic acid extraction efficiency between viruses and nucleic acid extraction kits have been observed before using serum, respiratory, faecal, and environmental samples [66,67,68,69,70]. Indeed, the extraction efficiency of some dsDNA phages is only 24–30% [23]. Structural proteins of the RNA viruses RV-A (VC2) and BVDV-1 (core) may inhibit extraction, which can be mitigated by the inclusion of proteases in the extraction kits [71,72]. The kit used here has no proteinases listed as a component of the lysis buffer and nucleic acid extraction kit [66], making it possible that the presence of viral core proteins could reduce the efficiency of viral nucleic acid recovery. Experimentally determined recovery efficiencies can be used to correct virus abundance in sequencing data [23], although based on our results, the question remains to what degree the recovery efficiency of one virus can be extrapolated to other viruses. The low recovery of eukaryotic viruses, particularly enveloped viruses MHV-68 and BVDV-1, may be due to the formation of aggregates and loss during centrifugation and/or due to the adsorption of faecal material to plasticware and filters used in their isolation leading to the loss of virus particles [14]. Indeed, two enveloped eukaryotic viruses, herpesvirus and coronavirus, were sensitive to centrifugation [12]. Another possible source of bias is the use of nucleases during the VLP extraction process, as some virus particles are susceptible to RNase [73]. DNA library preparation methods may also affect the representation of individual viruses, as WTA2 and SISPA show different relative abundances for several ssDNA and RNA virus taxa. The viruses of the phyla *Phixviricota* and *Cressdnaviricota*, as well as MC virus M13 (*Hofneiviricota*), are viruses with circular ssDNA genomes, and both M13 and faecal *Phixviricota* viruses have higher relative abundance in the SISPA than the WTA2 libraries of all three samples, as well as a large increase in *Cressdnaviricota* in one sample. Another striking difference is the nearly complete absence of RV-A in the SISPA libraries. While the SISPA libraries were produced several months after the WTA2 libraries, and thus, long-term storage of the nucleic acid extracts should be considered, this suggests that amplification of RV-A dsRNA by SISPA is less efficient than WTA2.

### 4.2. Effects of Sequence Bias

Sequence bias is a phenomenon of many sequencing methods [74]. Although both WTA2 and SISPA produce non-uniform coverage depths, the profiles are highly consistent in the WTA2 and SISPA libraries. While there are similarities in the depth profiles of WTA2 and SISPA, they ultimately produce different profiles, with higher peaks in some regions leading to reduced uniformity in SISPA (Figure 3A). This likely contributes to the higher assembly quality of the WTA2 libraries. With equivalent numbers of reads in the SISPA and WTA2 libraries, WTA2 yields more and longer contigs in the WTA2 library assemblies. Together with the fact that MC phages had more contigs in the MC HI SISPA libraries than the WTA2 libraries, with equal or lower coverage, this is indicative of higher fragmentation, which has been reported previously for SISPA [14]. Decreased uniformity of the sequence depth profiles means that certain regions of the genome are more likely to produce reads and contigs, and higher sequencing depth is required to fully recover the genome [75]. This may explain a higher fraction of contigs being identified as viral by geNomad in the WTA2 assemblies. Thus, a reduction in sequence bias and an increase in coverage depth uniformity will produce higher quality assemblies and enhance virus discovery.

Sequence bias is introduced by several factors that lead to preferential amplification of certain genomic regions. For instance, GC-content affects various DNA polymerases to various degrees [76], tag sequences and the random nucleotide length of tagged random primers and bases downstream of the binding site influence amplification [77,78], DNA polymerases have different sequence preferences [79], and the transposase used in the Illumina DNA Prep kit has a preferred sequence motif [80]. Optimisation of the WTA2 protocol is not straightforward due to the proprietary nature of kit components. Use of longer random nucleotide sequences in the tagged primers and using multiple primers with different tag sequences may increase uniformity of the coverage depth [77]. However, incorporation of multiple primers with twelve random nucleotides did not improve coverage depth uniformity in an oral virome study [15]. Our results suggest WTA2 produces more uniform profiles despite using random hexamers, while random octamers were used for SISPA. Nevertheless, optimisation of SISPA primers could be explored. WTA2 included 17 cycles of PCR amplification, while SISPA had 30 cycles, possibly amplifying any bias inherent in the method. For WTA2, increasing PCR cycles past 17 has been previously shown not to improve the yield of viruses [12] and a recent benchmarking study showed a reduced total assembly length when using 30 amplification cycles, compared to 15 or fewer cycles [30]. Thus, while it may require higher input volumes, a reduced number of cycles should be considered for SISPA.

### 4.3. Precision and Reliability of Abundance Measures

While most faecal viral sequences show large differences between the lowest and highest abundance across three replicates, most of these contigs had an individual relative abundance <0.1% and collectively made up between 1% and 10% of relative abundance in samples. This is likely because sequences with lower relative abundance have lower read counts and low-count data are inherently more noisy. Thus, high variability in these methods is restricted to a minority of sequences, and using a minimum coverage of 10X removed most of these sequences. Nonetheless, even for contigs with high coverage, some had >4-fold difference between the highest and lowest abundance across three replicates, and for contigs with at least 100X coverage, the median fold difference was still 1.5 and 1.2 on average across the three faecal samples in the WTA2 and SISPA libraries, respectively. SISPA libraries produced the most consistent abundance.

Relative abundance of virus sequences at the class level was consistent, except for *Phixviricota* in the SISPA library of sample S07. Only a small fraction of viral sequences could be assigned an order or family, preventing assessment of variability for those ranks. Although WTA2 assemblies have reduced fragmentation and increased diversity, the rate of classification at the order and family levels was similar for both SISPA and WTA2.

MC virus genomes were represented in the assemblies by several contigs that can have up to 30-fold difference in abundance. Theoretically, contigs in an assembly have a coverage of at least 1X, whereas the actual genome coverage can be much lower, leading to an overestimation of the abundance of the virus. Provided that contigs together sufficiently cover the virus genome (≥63%), the abundance is overestimated by up to only 10%, whereas a coverage of <32% leads to a doubling of the calculated abundance. The accuracy of the calculated virus abundance therefore benefits from grouping of contigs into genome bins that belong to the same virus, as well as estimation of the genome completeness. The former can be accomplished by virus genome binning tools like PHAMB [81], vRhyme [82], MetaBAT 2 [83,84], CoCoNet [85], and Phables [86], and the assembly contiguity improvement tool COBRA [87], whereas the latter can be performed by CheckV [52].

### 4.4. MC Spiking Concentration

The use of a virus MC has provided insights into the consistency of virus recovery across samples, biases in virus recovery, sequence bias, and accuracy and precision of calculated abundance. The spike concentration of MC HI was sufficient to recover full genomes for most dsDNA viruses in the assemblies of WTA2 libraries. However, SISPA libraries contained fewer reads, particularly for the smaller RNA viruses. Therefore, a higher spiking concentration is recommended, in the order of 1 × 10^8^ particles of each virus per 50 mg faeces. Alternatively, an MC in which virus concentrations are adjusted for the nucleotide content of the genome should in theory produce equal read numbers for each virus.

### 4.5. Recommendations

The data indicate that while both WTA2 and SISPA each have their biases, these biases are systematic and thus will allow valid comparisons of samples. Nonetheless, care should be taken when comparing samples, as more than two-fold differences between abundance measurements of sequences across three replicates were observed in more than 5% of contigs. For optimal precision of abundance measurement, contigs with a mean depth of at least 10X should be used, and sequences with at least 50% completeness are required for accurate estimation of abundance. Completeness can be achieved using various binning tools, with Phables previously producing the fewest chimeric and duplicate sequences, compared to VAMB [88] and vRhyme [89]. The choice between SISPA and WTA2 depends on the requirements of the researcher. Based on our results, SISPA is more cost effective and provides higher precision, while WTA2 provides higher assembly quality and diversity and thereby allows for improved virus discovery. During the writing of this manuscript, an alternative method was published based on the direct ligation of sequencing adapters to cDNA [29], which showed more accurate representation of DNA phages from a DNA phage MC, compared to MDA. Future comparison of this method to WTA2 and/or SISPA on DNA as well as RNA viruses will be valuable.

### 4.6. Limitations

While the WTA2 and SISPA libraries were generated from the same viral nucleic acid extracts, other sources of variation need to be considered. These include sample storage times, which for the SISPA libraries were longer, with Illumina libraries being prepared on separate occasions and sequenced on different sequencing platforms. An effect of each of these differences on the final data cannot be excluded. A sizeable portion of the sequencing data was attributable to sequences of unknown origin. These were especially pronounced in the SISPA libraries. Reads mapping to these contigs were enriched for bacterial reads, although a subset of these contigs may be viral sequences that were not recognised by geNomad. Additional virus detection tools, improved assembly contiguity and genome binning of contigs, and comparing non-recognised sequences to microbial and other sequence databases can help identify the source of these sequences. This would show whether SISPA libraries contain more contaminations, or whether the increased number of unclassified sequences is due to increased fragmentation hampering identification of virus sequences.

## 5. Conclusions

Using a set of faecal samples analysed in triplicate and spiked with a mock viral community, the reproducibility and bias of a virome sequencing method has been assessed, including the comparison of two methods for the reverse transcription and amplification of viral nucleic acid. The results show that individual viruses have different recovery efficiencies, and that recovery of individual viruses varies between replicates. Our comparison of WTA2 and SISPA shows that the WTA2 kit provides higher sensitivity, at the cost of higher variability. SISPA on the other hand is less sensitive and accurate but more consistent. While WTA2 is a proprietary kit, SISPA uses commonly used reagents and is thus less costly and less sensitive to supply chain issues since reagents can be substituted more easily. Future improvements to the SISPA protocol should be investigated to increase sensitivity, particularly to RNA viruses, as this would make SISPA a competitive alternative to WTA2. Incorporation of a mock viral community was key in this analysis and is a valuable control to be included in metagenomic studies [90,91]. The bioinformatics pipeline used in this study is available online.

## Figures and Tables

**Table 1:** Characteristics of the viral mock community.

Host	Virus	Species	Genome			Virion		MC	
	Name		Type	Top.	Size (kb)	Env.	Size (nm)	HI (VLP ^1^)	LO (VLP ^1^)
Prokar-yotic	Det7	*Kuttervirus Det7*	dsDNA	linear	157.5		90 ^C^/110 ^T^	1.0 × 10^7^	1.0 × 10^5^
	M13	*Inovirus M13*	ssDNA	circular	6.4		6.5 ^D^/ 860 ^L^	1.0 × 10^7^	1.0 × 10^5^
	P22	*Lederbergvirus P22*	dsDNA	linear	41.7		60	1.0 × 10^7^	1.0 × 10^5^
	T5	*Tequintavirus T5*	dsDNA	linear	121.7		90 ^C^/ 160 ^T^	1.0 × 10^7^	1.0 × 10^5^
Eukar-yotic	BVDV-1	*Pestivirus bovis*	ssRNA	linear	12.5	Yes	50	1.0 × 10^7^	1.0 × 10^5^
	MHV-68	*Rhadinovirus muridgamma4*	dsDNA	linear	119.5	Yes	220	2.5 × 10^6^	2.5 × 10^4^
	RV-A	*Rotavirus* *alphagastroenteritidis*	dsRNA	linear (11 Sg)	18.6		80	1.0 × 10^7^	1.0 × 10^5^

Env.: envelope, Sg.: genome segments, Top.: topology, ^1^ VLP per spike-in, ^C^ capsid size, ^T^ tail length, ^D^ virion diameter, ^L^ virion length.

**Table 2:** Overview of MC reference sequences used.

Virus	NCBI Accession	Sequence Name	Genome	GC%
Abbreviation	Accession Nr.		Bases	
BVDV	NC_001461.1	Bovine viral diarrhoea virus 1, complete genome	12,573	45.79
Det7	NC_027119.1	Salmonella phage Det7, complete genome	157,498	44.60
M13	NC_003287.2	Enterobacteria phage M13, complete genome	6407	40.74
MHV	NC_001826.2	Murine herpesvirus 68 strain WUMS, complete genome	119,451	47.23
P22	NC_002371.2	Salmonella phage P22, complete genome	41,724	47.09
RV-A	NC_011507.2	Rotavirus A segment 1, complete genome	3302	33.74
RV-A	NC_011506.2	Rotavirus A segment 2, complete genome	2693	32.97
RV-A	NC_011508.2	Rotavirus A segment 3, complete genome	2591	28.91
RV-A	NC_011510.2	Rotavirus A segment 4, complete genome	2362	34.67
RV-A	NC_011500.2	Rotavirus A segment 5, complete genome	1614	31.16
RV-A	NC_011509.2	Rotavirus A segment 6, complete genome	1356	38.57
RV-A	NC_011501.2	Rotavirus A segment 7, complete genome	1105	33.81
RV-A	NC_011502.2	Rotavirus A segment 8, complete genome	1059	33.30
RV-A	NC_011503.2	Rotavirus A segment 9, complete genome	1062	35.78
RV-A	NC_011504.2	Rotavirus A segment 10, complete genome	751	40.21
RV-A	NC_011505.2	Rotavirus A segment 11, complete genome	667	38.53
T5	NC_005859.1	Enterobacteria phage T5, complete genome	121,750	39.27

**Table 3:** MC virus reads in WTA2 and SISPA libraries.

Sample	Method	Total Reads (Million)			Mock Community Reads			Mock Community Reads per 10,000		
		HI	LO	No	HI	LO	No	HI	LO	No
S06	WTA2	14.43	10.38	13.51	313,522	3209	1	217	3.09	0.00
	SISPA	15.34	12.12	15.63	67,672	422	12	44	0.35	0.01
S07	WTA2	13.28	11.88	10.90	463,340	4168	12	349	3.51	0.01
	SISPA	10.38	11.98	12.27	182,285	649	9	176	0.54	0.01
S08	WTA2	12.34	9.66	10.06	478,236	1936	7	387	2.00	0.01
	SISPA	10.37	10.85	13.55	11,274	52	4	11	0.05	0.00
SMC	WTA2	9.23	4.20	-	4,271,871	67,654	-	4631	160.91	-
	SISPA	10.12	11.97	-	5,236,318	180,410		5174	150.75	-
SBL	WTA2	-	-	7.96	-	-	38,327	-	-	48.17
	SISPA	-	-	11.59	-	-	59,757	-	-	51.55

HI: high-concentration MC (1.25 × 10^9^ particles/g faeces), LO: low-concentration MC (1.25 × 10^7^ particles/g faeces), No: not-spiked sample, SISPA: sequence-independent single primer amplification, WTA2: whole transcriptome amplification kit, SMC: MC-only samples, SBL: blank samples.

## Data Availability

The virome metagenomic sequencing data cleaned using the cleanup pipeline are freely available under NCBI BioProject number PRJNA1189570. The code for the analysis pipeline used in this paper is available as a Nextflow pipeline at https://github.com/RHaagmans/mc-spike (accessed on 27 November 2024).

## Supplementary Materials

The following supporting information can be downloaded at: www.mdpi.com/article/10.3390/v17020155/s1, Figure S1: WTA2 and SISPA yield equivalent numbers of high-quality reads; Figure S2: SISPA libraries have a higher rate of duplicate reads than WTA2 libraries; Figure S3: Consistency of the sequencing depth profiles of virus genomes in spiked and MC-only samples processed using WTA2; Figure S4: Consistency of the sequencing depth profiles of virus genomes in spiked and MC-only samples processed using SISPA; Figure S5: Above 20% coverage, no relationship between difference in GC-content and coverage is detectable; Figure S6: Difference between GC-content of RV-A reads and genome segments differs by segment; Figure S7: A more uniform sequencing depth profile in WTA2 leads to improved genome coverage and lower overestimation of relative abundance; Figure S8: Contigs of unknown origin are enriched for bacterial reads; Figure S9: Most contigs with high variation are short contigs with low coverage; Figure S10: Variation in abundance-based ranking of contigs; Table S1: Number of reads per sample; Table S2: Assembly statistics for the WTA2 and SISPA libraries of spiked stool samples and MC-only samples; Table S3: Percentage of contigs for which the relative abundance (RPKM) of two randomly selected replicates falls within the a given percentage; Table S4: Variation in relative abundance at the phylum level between replicates of stool samples, based on individual replicate assemblies.
