# Supplementary material for: Assessing Bias and Reproducibility of Viral Metagenomics Methods for the Combined Detection of Faecal RNA and DNA Viruses
Source: Viruses. 2025 Jan 23;17(2):155. doi: 10.3390/v17020155 (PMC11860243; doi:10.3390/v17020155)
Supplement: Supplementary file 1 [file viruses-17-00155-s001.zip › viruses-3363932-supplementary.pdf]

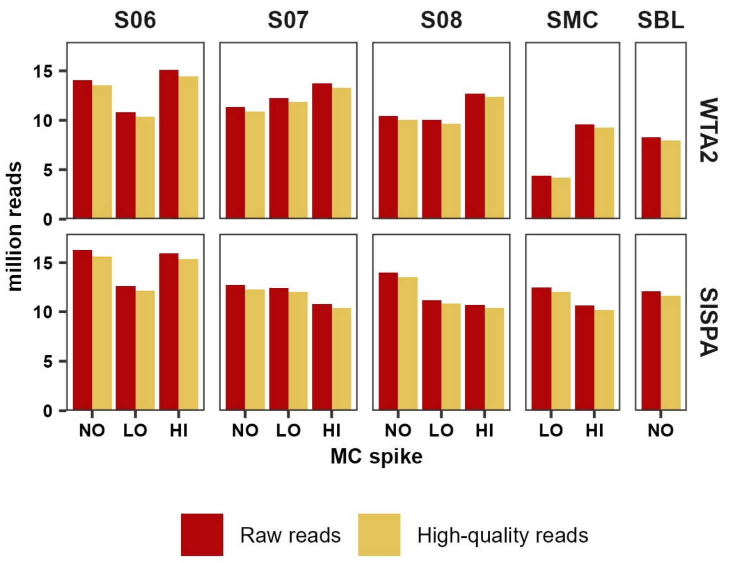

Figure S1: WTA2 and SISPA yield equivalent numbers of high-quality reads. The graphs depict million reads for stool sample aliquots and from mock-community-only and blank samples, processed using WTA2 and SISPA.

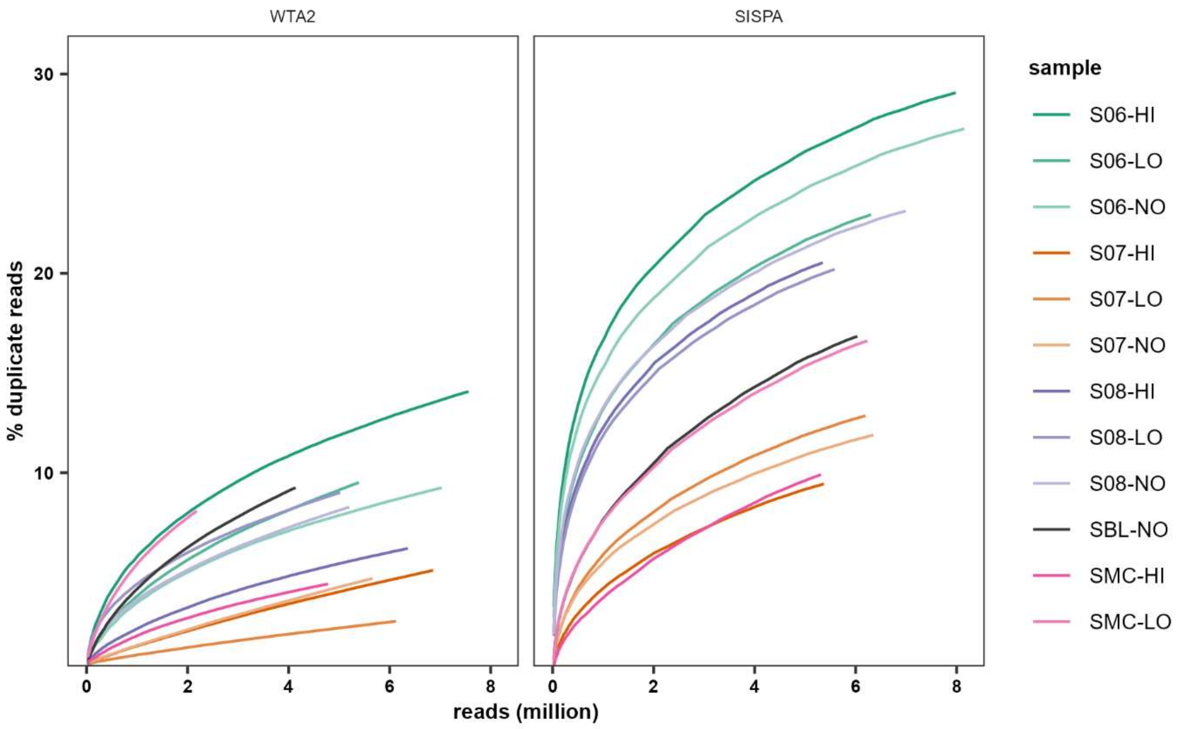

Figure S2: SISPA libraries have a higher rate of duplicate reads than WTA2 libraries.

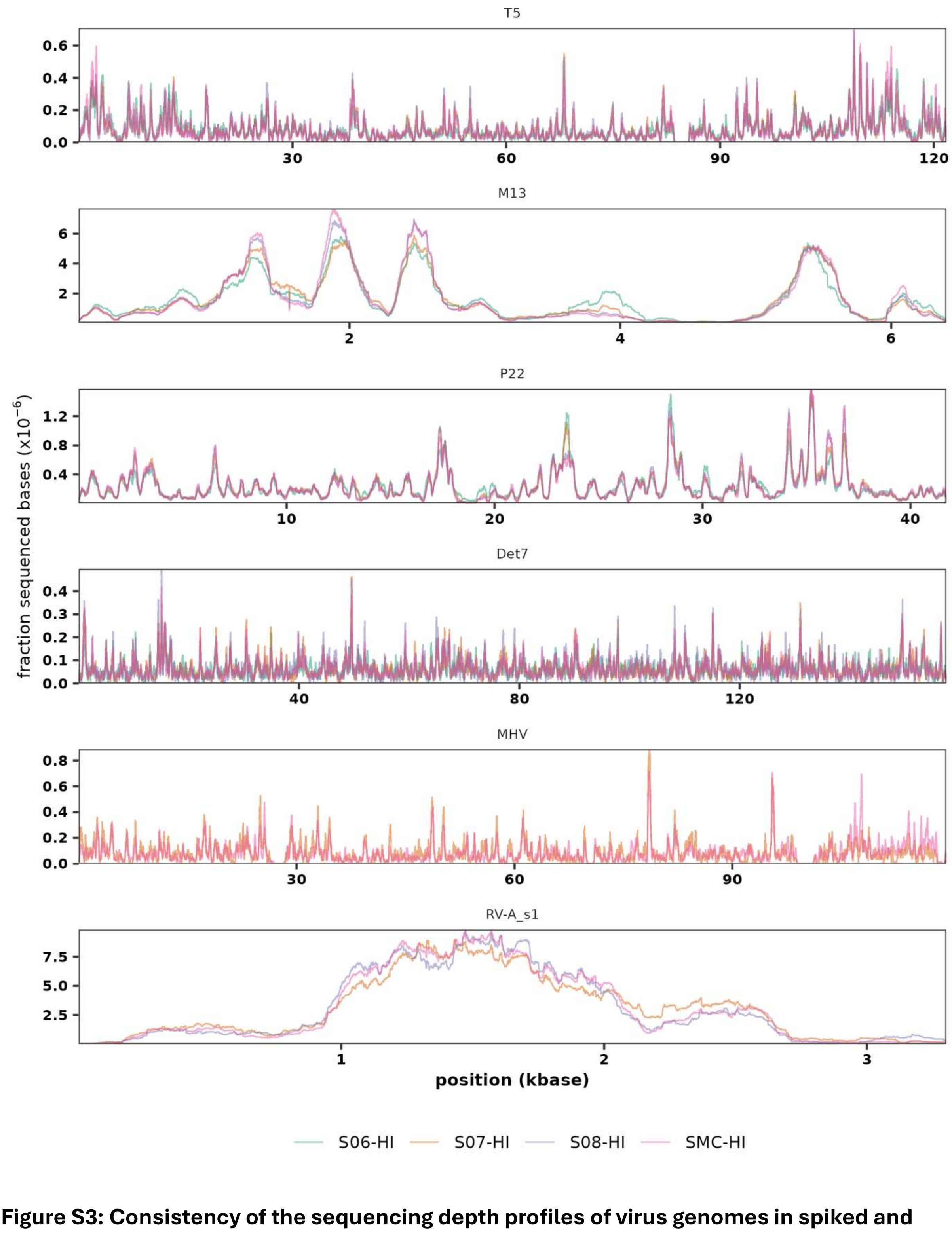

Figure S3: Consistency of the sequencing depth profiles of virus genomes in spiked and MC-only samples processed using WTA2. Sequencing depth, normalised by the total number of sequenced based in for each virus in each sample. Only data for samples with >80% genome coverage are displayed. For RV-A, only the depth of the first segment (RV-A_s1) is shown.

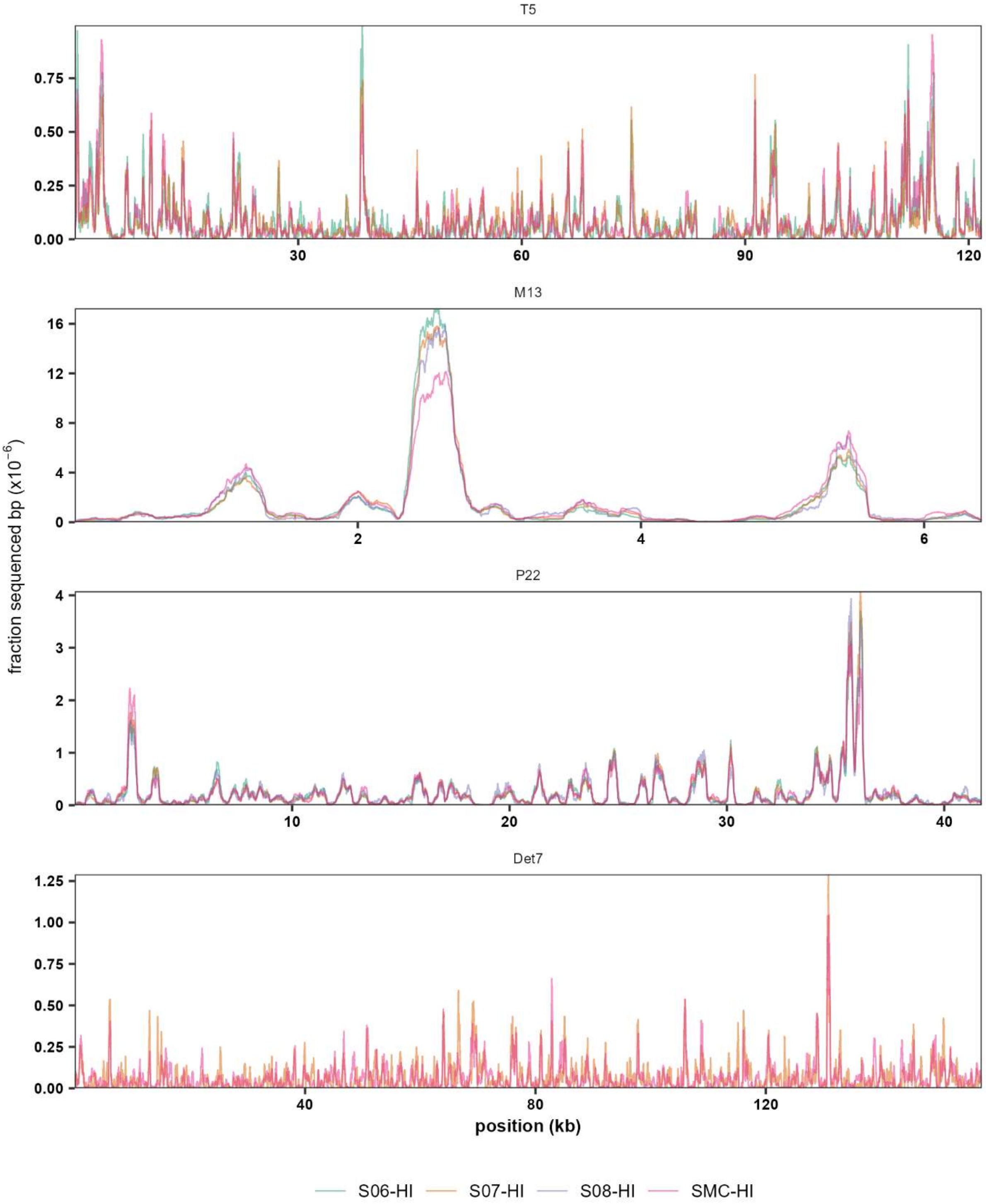

Figure S4: Consistency of the sequencing depth profiles of virus genomes in spiked and MC-only samples processed using SISPA. Relative sequencing depth, calculated as the fraction of sequenced bases for each virus in each sample mapping to each position. Only data for samples with >80% genome coverage are displayed.

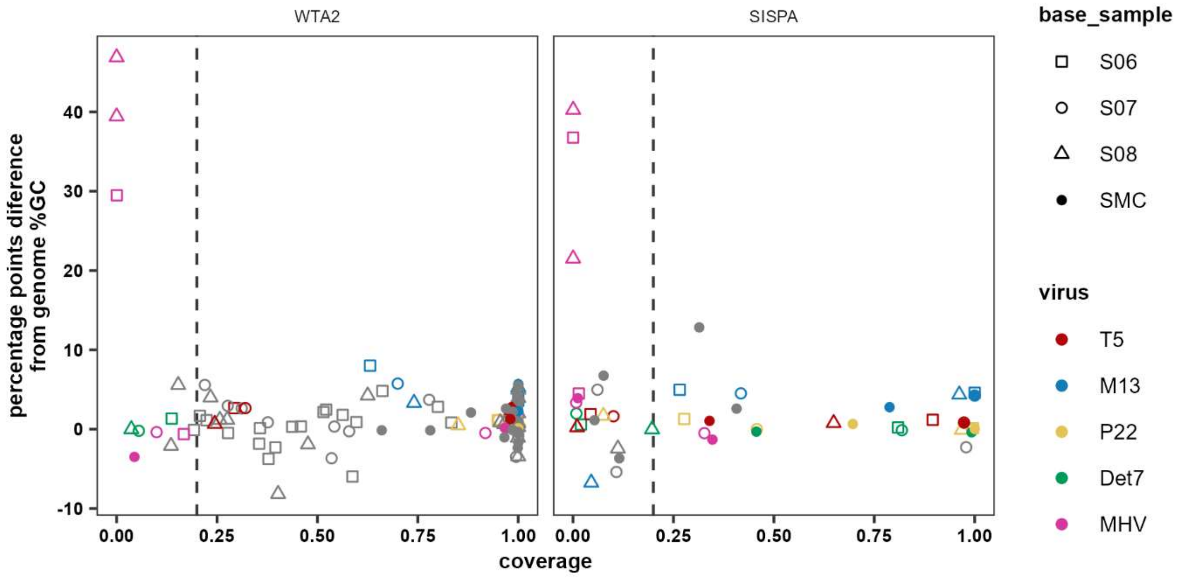

Figure S5: Above 20% coverage no relationship between difference in GC-content and coverage is detectable. The average GC-content of reads mapping to MC virus reference genomes was calculated and compared to the GC-content of the genome. The difference in percentage points between the average read GC-content and the actual genome GC-content is compared to the coverage of the genome of the virus in the sample. The dashed line depicts 20% coverage.

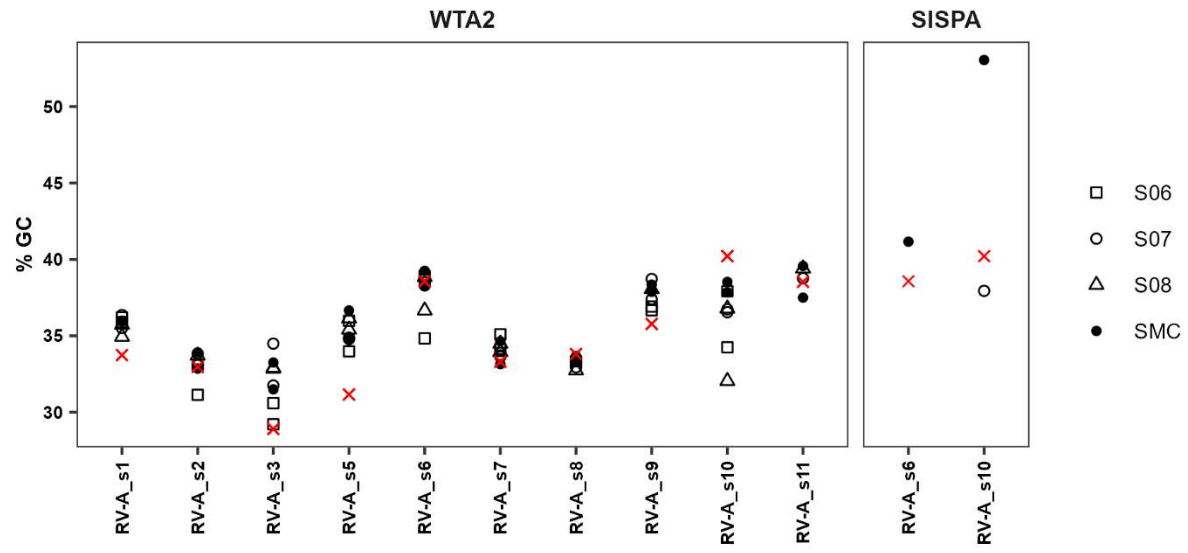

Figure S6: Difference between GC-content of RV-A reads and genome segments differs by segment. The average GC-content of reads mapping to RV-A reference genome segments was calculated and compared to the GC-content of the genome segments. The difference in percentage points between the average read GC-content and the actual genome segment GC-content is shown.

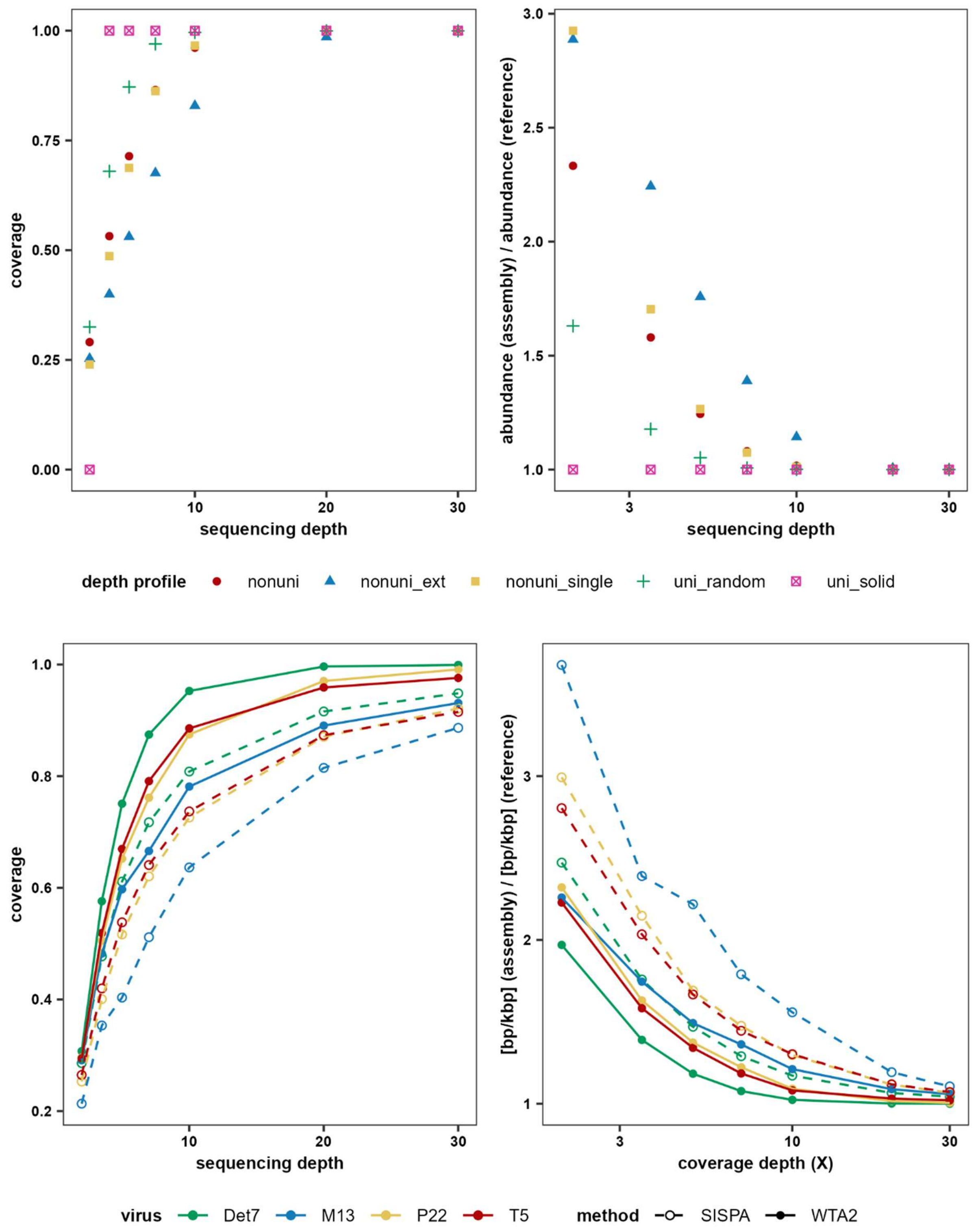

Figure S7: A more uniform sequencing depth profile in WTA2 leads to improved genome coverage and lower overestimation of relative abundance. A: Simulated coverage (left panels) and the ratio between reads in the assembly and the reference genome (right panels) for different sequencing depth profiles. In the top panels, first, the sequencing depth profile of a 100,000 bp genome is simulated. A non-uniform the depth profile is generated by adding four gaussian functions along the genome ("nonuni"). A second non-uniform profile consists of the same functions but higher peaks ("nonuni_ext"), and a third non-uniform profile profile consists

of a single gaussian function (“nonuni_single”). A uniform profile is generated with equal height for all positions. For the bottom panels, the coverage depth profiles of Det7, M13, P22, and T5 in the WTA2 and SISPA libaries are used. The profiles are used to assign weights to each position along the genome. A position along the genome is then randomly selected accoring to the weights, and a depth of 1 is added to the next 150 positions. This process is repeated for the number of reads required to achieve the desired overall sequencing depth. For a completely uniform sequencing depth profile (“uni_solid”), a fixed depth profile of the desired depth is generated in the top panels. Then, the number of sequenced bases based on the whole genome (reference), and assembly is calculated. Assembly is simulated, by selecting a minimum read depth of 2 and a minimum consecutive length of 300 bases for generating a contig. First a mask is created according to the minimum read depth, marking all positions below the minimum depth as zero and all above as one. Next, all sequences of ones shorter than the minimum length are changed to zero. Finally, the depth profile is multiplied by this mask to remove positions and corresponding depth numbers that would not be assembled.

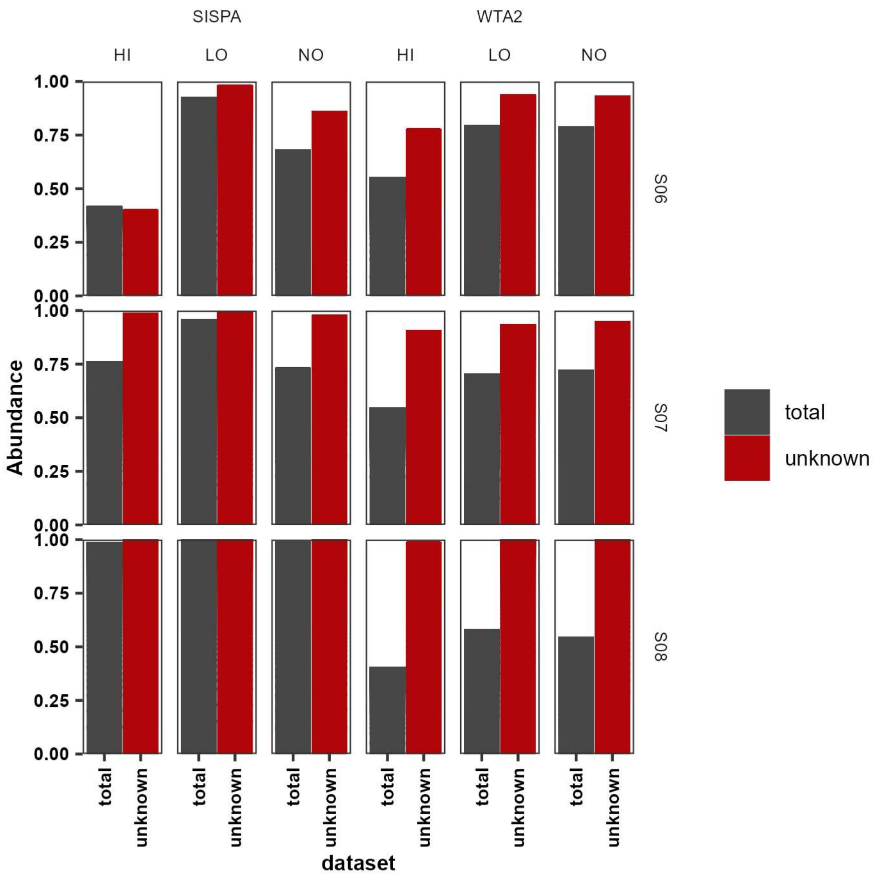

Figure S8: Contigs of unknown origin are enriched for bacterial reads. Total reads and reads mapping to contigs of unknown origin ("unknown") were mapped agains the Kraken2 Plus PFP database (04/09/2024) to determine the fraction of bacterial reads.

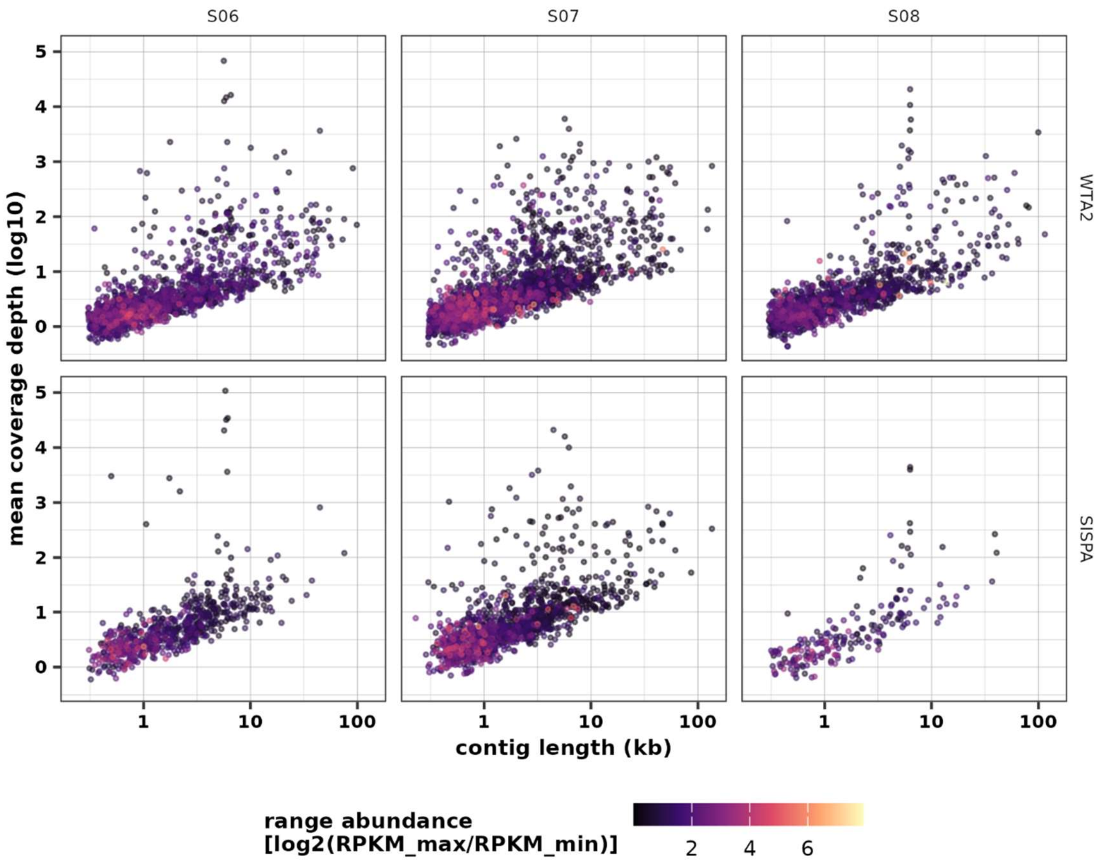

Figure S9: Most contigs with high variation are short contigs with low coverage. Mean coverage depth of virus contigs, calculated from the coverage depth of the three replicates, compared to the length of the contig. The colour scale shows the log2-transformed ratio between the highest and lowest abundance in RPKM.

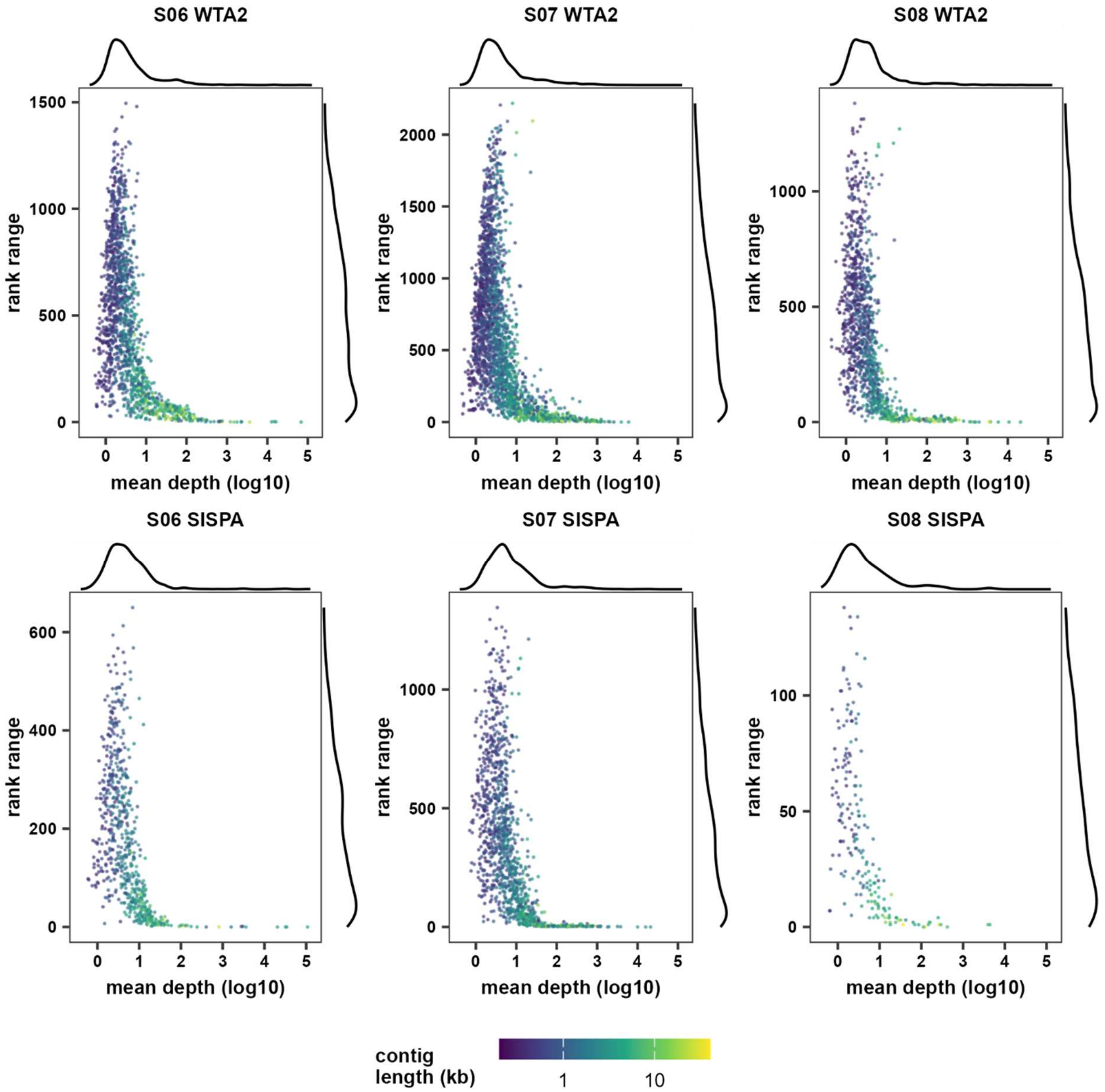

Figure S10: Variation in abundance-based ranking of contigs. The relative abundance is calculated for each contig and contigs are ordered according to their relative abundance to determine the abundance-based ranking. Only contigs present in all three replicates are included. The range in ranking shows the difference between the highest and lowest ranking of each contig. Since the maximum rank range depends on the number of contigs in each co-assembly, the y-axis is scaled differently for each sample. Graphs along the x- and y-axis show the density along the respective axis

Table S1: Number of reads per sample.

Sample	Spike	Method	Reads	Gb[1]	Mean read length	Mean read GC (%)
S06	HI	SISPA	15,965,882	2.314	144.95	46.420
S06	HI	WTA2	15,127,452	2.181	144.20	44.175
S06	LO	SISPA	12,609,288	1.813	143.75	48.385
S06	LO	WTA2	10,785,552	1.565	145.10	47.485
S06	No	SISPA	16,299,880	2.363	144.95	46.785
S06	No	WTA2	14,067,582	2.010	142.90	46.430
S07	HI	SISPA	10,743,842	1.566	145.75	46.135
S07	HI	WTA2	13,724,778	2.008	146.30	46.375
S07	LO	SISPA	12,396,394	1.786	144.05	48.795
S07	LO	WTA2	12,248,248	1.747	142.70	47.455
S07	No	SISPA	12,704,470	1.835	144.40	45.785
S07	No	WTA2	11,325,348	1.641	144.90	49.010
S08	HI	SISPA	10,700,552	1.511	141.25	51.070
S08	HI	WTA2	12,721,624	1.787	140.50	43.705
S08	LO	SISPA	11,168,354	1.563	139.95	51.860
S08	LO	WTA2	10,040,098	1.395	138.95	43.070
S08	No	SISPA	13,983,286	1.933	138.25	51.220
S08	No	WTA2	10,405,760	1.465	140.80	43.555
SBL	No	SISPA	12,070,126	1.758	145.60	49.505
SBL	No	WTA2	8,275,822	1.165	140.70	48.645
SMC	HI	SISPA	10,628,358	1.538	144.65	46.625
SMC	HI	WTA2	9,561,076	1.314	137.40	47.575
SMC	LO	SISPA	12,468,566	1.814	145.50	49.515
SMC	LO	WTA2	4,360,438	0.610	139.90	46.740
S06	HI	SISPA	15,965,882	2.314	144.95	46.420

[1]Gigabases

Table S2: Assembly statistics for the WTA2 and SISPA libraries of spiked stool samples and MC-only samples.

Sample	Spike	# contigs (≥10 kbp)	# contigs (x1000)	Total length (Mbp)	GC (%)	N50[1] (bp)	L50 (#)
**WTA2**							
S06	HI	206	24.9	22.0	43.76%	1,163	1,693
	LO	208	34.8	27.0	49.37%	857	2,205
	No	190	33.9	25.9	47.57%	836	2,175
S07	HI	270	54.1	43.0	49.29%	923	2,890
	LO	197	82.8	58.7	48.71%	749	3,296
	No	246	54.7	41.3	51.16%	819	2,858
S08	HI	91	19.5	16.2	48.17%	1,074	1,653
	LO	49	11.6	8.8	46.55%	858	1,118
	No	60	13.5	11.0	47.34%	973	1,280
SMC	HI	10	75.4	32.9	48.28%	411	1,315
	LO	13	36.8	16.3	42.63%	419	1,028
SBL	No	5	61.5	26.0	45.73%	400	1,207
**SISPA**							
S06	HI	10	12.2	8.5	45.71%	796	1,313
	LO	7	12.3	7.6	46.55%	646	1,243
	No	10	16.1	10.0	46.75%	642	1,477
S07	HI	78	37.0	23.3	47.15%	613	2,078
	LO	39	32.0	18.5	46.93%	572	1,757
	No	79	42.9	26.0	47.35%	576	2,169
S08	HI	6	3.6	2.1	47.20%	569	505
	LO	5	2.4	1.4	47.17%	586	379
	No	6	2.7	1.5	46.81%	577	419
SMC	HI	14	11.1	4.8	50.43%	396	533
	LO	1	8.9	3.7	51.78%	392	530
SBL	No	1	7.7	3.2	51.01%	391	499

[1]N50 is the length at which all contigs of that length or longer comprise 50% of the assembly;
[2]L50 is the minimum number of contigs in the assembly needed to collectively comprise at least 50% of the assembly;

Table S3: Percentage of contigs for which the difference in relative abundance (RPKM) of two randomly selected replicates is less within the given percentage.

		Maximum difference between two abundance values														
	**Mean CD[1]**	**10%**	**20%**	**30%**	**40%**	**50%**	**60%**	**70%**	**80%**	**90%**	**100%**	**110%**	**120%**	**130%**	**140%**	**150%**
**WTA2**	**LCD**	10.0%	19.5%	29.9%	40.1%	50.3%	58.8%	66.6%	73.0%	77.6%	80.5%	81.7%	82.8%	84.1%	85.5%	86.0%
	**HCD**	15.7%	33.4%	49.7%	62.5%	73.0%	79.2%	84.9%	89.4%	90.6%	92.2%	93.3%	93.9%	94.8%	95.6%	96.3%
**SISPA**	**LCD**	10.1%	21.8%	32.3%	42.8%	53.9%	62.2%	68.9%	74.4%	79.1%	82.0%	83.4%	84.4%	85.6%	86.6%	87.3%
	**HCD**	31.1%	52.3%	68.1%	77.1%	83.4%	88.2%	91.0%	92.4%	93.1%	94.7%	95.8%	96.2%	96.8%	97.3%	97.5%

[1] Mean coverage depth (LCD: <10X; HCD: ≥10X)

Table S4: Variation in relative abundance at the phylum level between replicates of stool samples, based on individual replicate assemblies.

		S06		S07		S08	
**Phylum**	**Method**	**Mean**	**Range[1]**	**Mean**	**Range[1]**	**Mean**	**Range[1]**
Phixviricota	WTA2	81.424%	6.221	22.722%	7.767	66.113%	2.948
Phixviricota	SISPA	97.047%	1.530	58.447%	30.439	75.178%	9.748
Uroviricota	WTA2	18.336%	5.990	75.118%	6.223	33.207%	3.090
Uroviricota	SISPA	2.857%	1.408	32.577%	24.007	24.697%	9.858
Kitrinoviricota	WTA2	0.029%	0.032	0.279%	0.396	0.074%	0.074
Kitrinoviricota	SISPA	0.027%	0.040	4.089%	4.307	0.041%	0.097
Cressdnaviricota	WTA2	0.067%	0.043	0.553%	0.270	0.158%	0.065
Cressdnaviricota	SISPA	0.014%	0.003	4.326%	1.810	0.028%	0.047
Pisuviricota	WTA2	0.065%	0.109	0.877%	1.436	0.007%	0.020
Pisuviricota	SISPA	0.027%	0.052	0.069%	0.043	0.000%	0.000

[1]Range is the difference in percentage points between the highest and lowest relative abundance of three replicates.
